# Homogenizing out-of-plane strain distribution for high-performance flexible perovskite photovoltaics

**DOI:** 10.1126/sciadv.aec3238

**Published:** 2026-04-10

**Authors:** Yang Zhong, Xiao Luo, Binlou Gao, Xueying Wang, Gengling Liu, Wangping Sheng, Zhiwei Ren, Gang Li, Licheng Tan, Yiwang Chen

**Affiliations:** ^1^ College of Chemistry and Chemical Engineering/Film Energy Chemistry for Jiangxi Provincial Key Laboratory (FEC)/Institute of Polymers and Energy Chemistry (IPEC), Nanchang University, 999 Xuefu Avenue, Nanchang 330031, China.; ^2^ College of Chemistry and Materials Science, Gannan Normal University, Ganzhou 341000, China.; ^3^ College of Chemistry and Chemical Engineering, Jiangxi Province Engineering Research Center of Ecological Chemical Industry, Jiujiang University, Jiujiang 332005 China.; ^4^ Advanced Materials and Electronics Laboratory, Department of Electrical and Electronic Engineering, The Hong Kong Polytechnic University, Hong Kong SAR, China.; ^5^ Key Laboratory of Fluorine and Silicon for Energy Materials and Chemistry of Ministry of Education, Jiangxi Normal University, 99 Ziyang Avenue, Nanchang 330022, China.

## Abstract

Flexible perovskite solar cells (PVSCs) are promising for next-generation photovoltaic due to their lightweight and flexibility. However, nonuniform out-of-plane strain from heterogeneous A-site doping and residual stress from PbI_2_-rich surfaces limits their long-term stability and mechanical robustness. Here, we demonstrate that optimized A-site doping reduces defect density and microstrain, improving compositional homogeneity. Advanced visualization of out-of-plane strain reveals key pathways for strain homogenization. Additionally, an in situ–formed 2D perovskite layer on PbI_2_-rich surface effectively relieves residual stress, promotes interfacial carrier transport, and strengthens the mechanical property of perovskite film. Consequently, we achieve champion power conversion efficiencies of 26.59% for rigid and 25.88% (certified 25.55%) for flexible PVSCs. Furthermore, large-area flexible modules obtain impressive efficiencies of 21.77% (25 square centimeters) and 19.23% (100 square centimeters). Unencapsulated flexible devices retain 97.8% initial efficiency after 2000 hours of operation tracking (ISOS-L-1) while also demonstrating outstanding durability in damp-heat, thermal cycling, and mechanical tests. This work provides critical foundation for advancing the commercialization of flexible PVSCs.

## Teaser

Strain homogenization and in situ 2D layer enable flexible solar cells with high efficiency and bending endurance.

## INTRODUCTION

Lead halide perovskite (APbX_3_) has emerged as a transformative material for photovoltaic applications, owing to its exceptional optoelectronic properties, including high absorption coefficient, long carrier lifetime, and superior defect tolerance (*1*–*5*). The lightweight construction, mechanical flexibility, and low-temperature solution processability make perovskite absorber layer particularly well suited for flexible perovskite solar cells (PVSCs) (*6*–*9*), enabling applications in portable power sources, wearable electronics, building-integrated photovoltaics, and space technologies (*10*–*13*). These advantages address many of the limitations inherent to traditional rigid crystalline silicon solar cells. However, the commercialization of flexible PVSCs remains constrained by challenges related to long-term stability and mechanical durability, largely stemming from lattice strain in the soft perovskite framework and residual stress at grain boundaries and surfaces (*14*, *15*). Particularly detrimental is the out-of-plane strain—deformation perpendicular to the substrate—which arises from thermal expansion mismatch and concentrates under bending, driving defect formation and mechanical failure (*15*, *16*). Homogenization of this strain is therefore crucial for robust flexible devices.

The crystal structure of organic-inorganic hybrid perovskite consists of corner-sharing [PbX_6_]^4−^ octahedra forming a three-dimensional (3D) cubic octahedral framework, wherein A-site cation doping [e.g., formamidinium (FA^+^) and methylammonium (MA^+^)] plays a pivotal role in governing lattice stability and optoelectronic performance (*17*–*20*). Although mixed-cation strategies, such as FA/MA codoping, are widely used to enhance phase stability and efficiency, the size mismatches among cations often induce lattice distortion and microstrain, resulting in structural disorder and increased defect densities (*3*, *21*–*23*). These microstructural issues not only compromise mechanical robustness but also accelerate device degradation under operational stressors such as illumination and thermal cycling. Recent advances in A-site cation and interface engineering have shown promise in mitigating lattice strain and improving device performance (*24*–*29*). For instance, Kim *et al.* (*26*) have introduced oversized methylenediammonium (MDA^2+^) cations to relieve strain within α-FAPbI_3_ lattice, thereby enhancing both efficiency and stability. Nevertheless, systematic investigations into the influence of A-site cation selection on microstrain distribution, particularly the uniformity of out-of-plane strain, remain limited. Furthermore, incomplete crystallization often results in residual lead iodide (PbI_2_) at grain boundaries and surfaces, exacerbating residual stress and impairing charge transport (*30*). 2D perovskites with tailored structures have been demonstrated to effectively optimize interfacial carrier dynamics without sacrificing efficiency (*31*–*35*). Their intrinsic mechanical robustness further shields the bulk perovskite from environmental degradation and mechanical stress (*36*–*40*). Therefore, the design of 2D interfacial capping layer capable of in situ–passivating PbI_2_ while simultaneously maintaining high carrier mobility and mechanical resilience is critical for advancing flexible PVSCs.

**Fig. 1.**
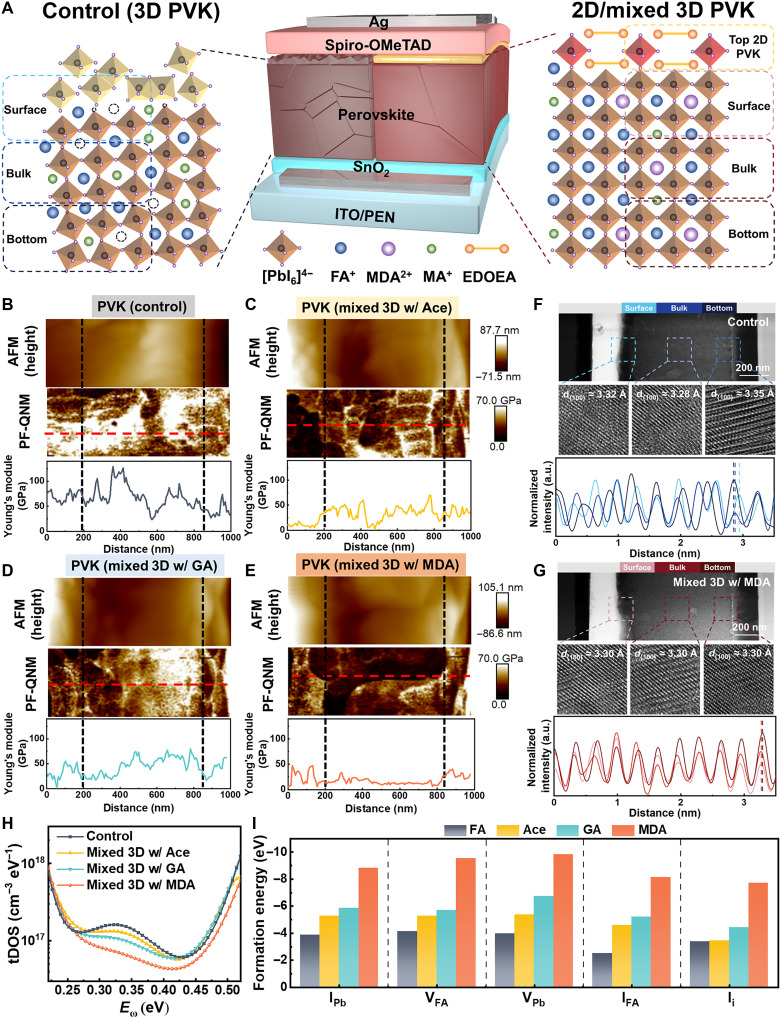
Strain engineering and defect suppression in mixed-cation perovskite films. (**A**) Schematic illustration of strain homogenization mechanism achieved through A-site cation alloying and incorporation of top 2D perovskite layer. (**B** to **E**) Nanomechanical mapping of perovskite devices: (top) AFM topography, (middle) peak force quantitative nanomechanical mapping (PF-QNM) modulus images, and (bottom) Young’s modulus difference profiles, comparing control and various A-site cation-mixed perovskite films. (**F** and **G**) Atomic-scale strain analysis: (top) high-angle annular dark-field scanning transmission electron microscopy cross sections of ITO/SnO_2_/perovskite/Spiro-OMeTAD/Ag/Pt stacks, (middle) atomic-resolution TEM images (scale bar, 7.8 nm) with measured interplanar spacings, and (bottom) corresponding intensity line profiles quantifying lattice distortions. (**H**) Trap density of state (tDOS) plots extracted from thermal admittance spectroscopy of the devices based on the control and various cations-doped perovskite films, where *E*_ω_ represents the characteristic energy corresponding to the frequency response. (**I**) Calculated defect formation energies of various cations-doped perovskites, focusing on negatively charged defects including iodine-lead antisites (I_Pb_), formamidinium vacancies (V_FA_), lead vacancies (V_Pb_), iodine-formamidinium antisites (I_FA_), and iodine interstitials (I_i_). a.u., arbitrary units.

Herein, we have systematically investigated the role of A-site cation doping in regulating lattice microstrain distribution (Fig. 1A). By optimizing cation composition, we effectively suppress defect densities and microstrain associated with cation alloying, achieving uniform out-of-plane strain profiles. Cross-sectional peak force quantitative nanomechanical mapping (PF-QNM) enables direct visualization and quantification of Young’s modulus gradients across the perovskite layer, thereby elucidating strain homogenization pathways. To address the detrimental effects of residual PbI_2_, we have introduced a diammonium spacer that in situ reacts with PbI_2_, forming vertically aligned 2D perovskite interlayer. This interface engineering simultaneously releases residual stress, enhances interfacial carrier transport, and improves mechanical toughness of perovskite film. As a result, rigid and flexible PVSCs achieve a champion efficiency of 26.59 and 25.88% (certified 25.55%, flexible), with unencapsulated device maintaining 97.8% initial efficiency after 2000 hours of continuous operation under maximum power point (MPP) tracking (ISOS-L-1 protocol). Encapsulated devices demonstrate outstanding long-term stability, retaining approach 90% after 1000 hours of damp-heat testing [85°C/85% relative humidity (RH)] following the ISOS-D-3 protocol and 92.1% of initial efficiency after 500 hours of thermal cycles (−40° to 85°C). Notably, the combination of uniform strain distribution and robust interfacial structure confers exceptional mechanical resilience, with the device retaining more than 90% of its initial efficiency after 11,000 bending cycles. This work elucidates fundamental principles of strain engineering and interfacial stress-release, providing critical insights for the commercialization of flexible perovskite photovoltaics.

## RESULTS

### Strain engineering and defect control in perovskites

Building upon established cation engineering strategies, we have systematically investigated three A-site cations with distinct steric and electronic characteristics: (i) acetamidinium (Ace^+^; *r* = 2.77 Å), FA^+^ analog extended by a methyl group; (ii) guanidinium (GA^+^; *r* = 2.78 Å), featuring an amino-functionalized structure; and (iii) methylenediammonium (MDA^2+^; *r* = 2.62 Å), bearing two positive charges (fig. S1). These cations have substantially larger ionic radii than conventional FA^+^ (2.53 Å) and MA^+^ (2.17 Å), enhancing lattice interactions via both steric effects and electrostatic modulation. Compositional screening reveals an optimal doping concentration of 3 mol % for all cations, with MDA^2+^-incorporated devices demonstrating superior crystallinity, optoelectronic properties, and photovoltaic performance (figs. S2 to S6).

We have systematically investigated the mechanical properties of perovskite films with different A-site cation mixtures using peak force quantitative nanomechanical atomic force microscopy (AFM). As shown in fig. S7, the methylenediamine dihydrochloride (MDA)–mixed perovskite film displays the lowest average Young’s modulus (2.34 GPa), whereas the control, acetamidinium hydrochloride (Ace)–mixed, and guanidinium chloride (GA)–mixed films exhibit moduli of 8.54, 6.50, and 3.16 GPa, respectively. This trend has been independently verified through nanoindentation measurements (note S1, fig. S8, and table S1), which yielded higher absolute moduli while confirming the same relative order of mechanical flexibility across the sample series (*41*). The difference in absolute values stems from the distinct probing mechanisms: Nanoindentation reflects bulk, plastic-yield properties, while PF-QNM is sensitive to near-surface, elastic-viscoelastic response. To probe local mechanical properties with high spatial resolution, we have used cross-sectional PF-QNM (fig. S9 and Fig. 1, B to E). Operating in tapping mode, this advanced technique enabled unprecedented visualization of intramembrane mechanical heterogeneity. The control film exhibits substantial spatial fluctuations in the measured Young’s modulus. Extreme values within the ~100 GPa range are attributed to the localized probing of high-modulus underlying layers [e.g., indium tin oxide (ITO) and SnO_2_] or residual PbI_2_. These fluctuations are accentuated by the film’s rough and inhomogeneous morphology. Ace- and GA-doped films reduced this disparity to ~50 GPa, while MDA-doped film achieved near-perfect homogeneity with variations confined to ~10 GPa, fundamentally improving strain distribution uniformity. We have further conducted Williamson-Hall analysis to quantitatively assess intrinsic lattice microstrain (fig. S10 and note S2) (*42*, *43*). MDA-incorporated film exhibits the lowest microstrain (ε = 0.30 × 10^−3^), representing a 4.5-fold reduction compared to control film (ε = 1.35 × 10^−3^) and substantially lower than Ace- (ε = 1.15 × 10^−3^) and GA-modified films (ε = 0.77 × 10^−3^). Depth-resolved measurements revealed exceptional strain homogeneity, correlating well with PF-QNM mapping and indicating a substantial reduction in microstrain and a more uniform distribution of mechanical strain. As illustrated in figs. S11 and S12, the strategic incorporation of MDA^2+^ with its optimal ionic radius and Goldschmidt tolerance factor enables simultaneous mitigation of intrinsic lattice strain while maintaining structural integrity, thereby enhancing both mechanical flexibility and optoelectronic stability-critical requirements for operational device reliability.

To elucidate the strain homogenization induced by MDA incorporation, we have conducted cross-sectional high-angle annular dark-field scanning transmission electron microscopy (TEM) to quantitatively analyze interplanar spacings (*d*) across surface, bulk, and bottom regions (Fig. 1, F and G). In the control sample, *d*_{100}_ varies notably (*d*_surface_ = 3.32 Å, *d*_bulk_ = 3.28 Å, and *d*_bottom_ = 3.35 Å), whereas MDA-doped film exhibits uniform spacing (3.30 Å across all regions). This finding underscores the critical role of MDA-doping in compensating for the lattice strain induced by FA^+^ and MA^+^ size disparities, thereby achieving a homogenized out-of-plane strain distribution. Time-of-flight secondary ion mass spectrometry (TOF-SIMS) measurement (fig. S13) further reveals a homogeneous MDA distribution, consistent with modulus mapping, affirming the critical role of MDA in achieving uniform stress distribution. Furthermore, density functional theory (DFT) calculations elucidate the influence of cation incorporation on lattice parameters (fig. S14). The strength of hydrogen bonding between cations and [PbX_6_]^4−^ cages critically affects Pb─I bond lengths and Pb─I─Pb bond angles, which directly determine the lattice microstrain in perovskites (*17*). MDA-doped perovskite exhibits the highest binding energy (−7.87 eV/nm^2^), optimal Pb─I─Pb bond angle (~164.08°), and appropriate Pb─I bond length (~3.222 Å), collectively contributing to moderate lattice expansion without inducing excessive distortion and thus effectively relieving lattice strain.

Defect formation, another critical factor influencing device performance, has been investigated using thermal admittance spectroscopy and space-charge–limited current measurements (Fig. 1H, fig. S15, and note S3) (*44*, *45*). The results demonstrate that MDA-doped perovskite film exhibits substantially reduced density of both shallow and deep-level defects, along with a marked decrease in trap-filling limit voltage (*V*_TFL_). This phenomenon likely stems from the uniform stress distribution increasing defect formation energies, thereby suppressing vacancy defect formation. To further validate these findings, we have performed DFT calculations to determine formation energies for various defects, including iodine-lead antisites (I_Pb_), formamidinium vacancies (V_FA_), lead vacancies (V_Pb_), iodine-formamidinium antisites (I_FA_), and iodine interstitials (I_i_) in different cation-doped systems (fig. S16). As shown in Fig. 1I, MDA doping substantially increases formation energies for all defect types, particularly for V_Pb_ and V_FA_. Furthermore, temperature-dependent x-ray diffraction (XRD) analysis reveals that the MDA-doped film exhibits markedly reduced thermal lattice strain and a lower Young’s modulus compared to the control (fig. S17 and note S4), corroborating that the homogenized, compliant lattice effectively mitigates stress accumulation (*46*, *47*). These computational results agree well with experimental observations, confirming that MDA doping effectively regulates lattice parameters, homogenizes strain distribution, and suppresses defect formation—key factors that underpin the enhanced mechanical stability and, as demonstrated in later sections, the superior operational stability of the resulting devices.

### Characterization of stress-relief 2D perovskite layer

**Fig. 2.**
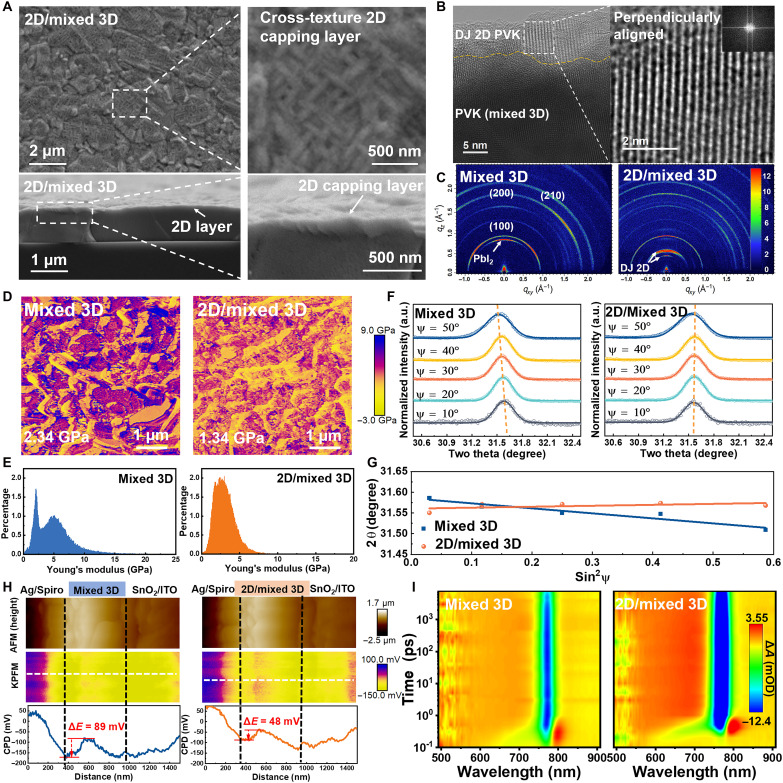
Preparation and characterization of stress-relief 2D perovskite layer. (**A**) Top-view and cross-sectional SEM images of 2D/mixed 3D perovskite films, illustrating film morphology and layered structure. (**B**) Cross-sectional high-resolution TEM image near the hole transport layer interface, a magnified inset highlighting the perpendicular orientation of the Dion-Jacobson phase 2D perovskite. (**C**) Grazing-incidence wide-angle x-ray scattering (GIWAXS) patterns of mixed 3D and 2D/mixed 3D perovskite films, showing crystallographic differences induced by the top 2D layer. (**D** and **E**) Surface mechanical properties and distribution characterized by PF-QNM modulus measurements for both mixed 3D and 2D/mixed 3D perovskite films. (**F** and **G**) Depth-dependent GIXRD patterns acquired at varying incident angles (F) and the linear fit 2θ-sin^2^(ψ) (G) for mixed 3D and 2D/mixed 3D perovskite films, revealing structural variations through the film depth. (**H**) KPFM measurements of devices based on mixed 3D and 2D/mixed 3D perovskite films, mapping potential distributions across the layers. (**I**) Pseudocolor femtosecond transient absorption spectroscopy (fs-TA) spectra of mixed 3D and 2D/mixed 3D perovskite films deposited on quartz substrates, capturing carrier dynamics.

To construct an in situ stress-relief layer on the surface of 3D perovskite films, we have strategically introduced three distinct diammonium spacer cations: alkane-based octane diammonium (lacking heteroatoms), oxygen-containing ethylenedioxyethane diammonium (EDOEA), and aromatic benzene-based *p*-phenylenediammonium (BDA). Perovskite devices incorporating these cations have been fabricated, with EDOEA demonstrating the most favorable performance metrics (fig. S18). Consequently, EDOEA has been selected as the primary candidate for further investigations aimed at addressing excess PbI_2_ residue and the associated residual stress in MDA-mixed perovskite films, which arise from incomplete crystallization, and for constructing a stress-release 2D perovskite layer through in situ reaction. Surface and cross-sectional scanning electron microscopy (SEM) characterization reveals the formation of a cross-textured DJ-type 2D perovskite capping layer, resulting from the reaction between PbI_2_ and EDOEA on the 3D perovskite surface (fig. S19 and Fig. 2A). This unique “patch-like” morphology effectively repairs grain boundary defects and suppresses mechanical fracture propagation under external stress, thereby notably enhancing the mechanical toughness of the perovskite film, as confirmed by quantitative double cantilever beam fracture toughness measurements (see note S5 and fig. S20) (*48*, *49*).

Further structural characterization using TEM confirms the successful formation of vertically oriented 2D perovskite layers on 3D perovskite surfaces, with excellent lattice matching and favorable lattice spacing between the 2D and 3D perovskites, which facilitates the formation of a coherent interface (Fig. 2B and fig. S21). Grazing-incidence wide-angle x-ray scattering (GIWAXS) patterns show additional diffraction peaks in the low-*q* region for 2D heterojunctions, along with nearly complete disappearance of PbI_2_ characteristic peaks in mixed 3D perovskite films (Fig. 2C and fig. S22). These observations demonstrate that in situ–grown 2D perovskite not only repairs the 3D perovskite surface but also markedly improves vertical crystal orientation, thereby verifying the successful construction and reconstruction effect of the 2D perovskite layer. Furthermore, x-ray photoelectron spectroscopy (XPS) analysis reveals chemical state changes in 2D/mixed 3D perovskite (fig. S23), showing notably reduced binding energies for Pb 4f and I 3d with complete disappearance of metallic Pb^0^ signals. This indicates complete conversion of surface PbI_2_ to 2D perovskite and strong interfacial coupling between 2D and 3D perovskites (*50*). Such strong interaction not only promotes vertical growth of 2D perovskite but also inhibits film degradation, substantially enhancing stability.

The mechanical properties of the stress-release 2D perovskite layer have been further investigated by PF-QNM measurements (Fig. 2, D and E, and fig. S24). The results reveal a substantially lower Young’s modulus at grain boundaries compared to that within grains in 3D perovskite, indicating that mechanically weak regions are susceptible to crack initiation. In contrast, the 2D/mixed 3D perovskite exhibit a reduced surface-localized Young’s modulus (1.34 GPa versus 2.34 GPa for the mixed 3D), which further reflects enhancement in mechanical toughness through grain boundary defect passivation and homogenization of interfacial stress. Nanoindentation has independently confirmed this softening trend (fig. S25). To probe the macroscopic residual strain, we have used complementary depth-dependent grazing-incidence x-ray diffraction (GIXRD) analysis (Fig. 2, F and G, and note S6), which reveals a notable reduced slope for the 2D/mixed 3D perovskite, indicating a homogenization of the near-surface strain field (*43*, *51*, *52*). This transition from a residual tensile strain to a more uniform and compressive state is attributed to the synergistic effect of interfacial lattice matching and stress redistribution by the soft 2D layer. This engineered compressive strain state enhances the perovskite lattice, suppresses defect formation, and enhances resistance to crack propagation, collectively improving mechanical robustness and stability of the device (*16*, *45*).

Systematic optical and electronic characterizations, including photoluminescence (PL), time-resolved photoluminescence (TRPL), and PL mapping, show markedly enhanced PL intensity and longer carrier lifetimes in 2D/mixed 3D perovskite films, indicative of improved charge transport and suppressed nonradiative recombination (fig. S26). The uniform PL intensity distribution across the films confirms reduced defect densities throughout the film. Surface Kelvin probe force microscopy (KPFM) measurements reveal more uniform and higher surface potential in 2D/mixed 3D perovskite film (fig. S27), demonstrating reduced surface trap states and improved charge extraction efficiency. Cross-sectional KPFM with contact potential difference (CPD) analysis (Fig. 2H) shows smoother potential distribution (Δ*E* = 48 mV) in the 2D/mixed 3D perovskite film, indicative of enhanced charge transport pathways and reduced energy barriers (*53*). Moreover, transient absorption (TA) spectroscopy (Fig. 2I and fig. S28) demonstrates prolonged decay kinetics in 2D/mixed 3D perovskite film, further confirming suppressed nonradiative losses and improved charge separation efficiency (*54*, *55*). Collectively, these comprehensive characterizations systematically demonstrate the advantages of 2D/mixed 3D perovskite film in carrier transport, surface electronic properties, and optoelectronic performance. The in situ–constructed 2D capping layer markedly improves film quality and device stability through effective surface passivation, optimization of charge distribution, and suppression of nonradiative recombination, thus providing critical theoretical and technical support for the development of high-performance flexible PVSCs.

### Performance and stability of flexible solar cells

**Fig. 3.**
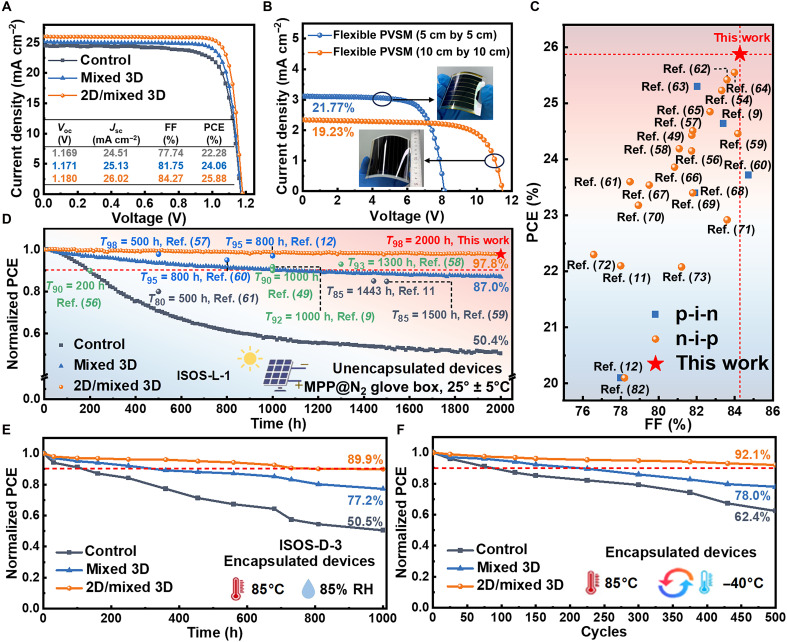
Photovoltaic performance and stability of flexible PVSCs. (**A**) *J-V* curves of champion flexible PVSCs based on control, mixed 3D, and 2D/mixed 3D perovskite films. (**B**) *J-V* curves of large-area (5 cm by 5 cm and 10 cm by 10 cm) flexible PVSM based on 2D/mixed 3D perovskite film. (**C**) Statistical diagram of power conversion efficiency (PCE) and fill factor (FF) values obtained from recently reported flexible PVSCs. (**D**) Normalized evolution of PCE and summary of the recently reported for unencapsulated flexible PVSCs under continuous tracking at the MPP following the ISOS-L-1 protocol. (**E**) Normalized evolution of PCE for encapsulated flexible PVSCs under damp-heat conditions (85°C and 85% RH) following the ISOS-D-3 protocol. (**F**) Normalized PCE evolution for encapsulated flexible PVSCs during thermal cycling tests from −40° to 85°C. h, hours.

To validate the performance enhancement from optimized out-of-plane strain distribution and the introduction of stress-release layers in flexible PVSCs, we have fabricated corresponding devices and systematically characterized their optoelectronic properties. Current density–voltage (*J-V*) curves champion rigid (fig. S29) and flexible (Fig. 3A) PVSCs, and corresponding photovoltaic parameters (tables S4 and S5) reveal notable performance differences among three device configurations. Notably, the 2D/mixed 3D device achieves a champion power conversion efficiency (PCE) of 26.59%/25.88% (rigid/flexible), significantly outperforming mixed 3D (25.20%/24.06%) and control devices (23.34%/22.28%). The negligible *J-V* hysteresis with an index of 0.0089 (fig. S30) and narrow PCE distribution (fig. S31) highlight efficient charge extraction and superior reproducibility, attributed to optimized film quality and homogeneity. Moreover, large-area (5 cm by 5 cm and 10 cm by 10 cm) flexible perovskite solar modules (PVSMs) based on 2D/mixed 3D achieve a PCE of 21.77 and 19.23% (Fig. 3B and table S6), demonstrating the practical scalability of the approach. The champion flexible PVSC and PVSMs demonstrate PCEs among the highest reported to date (Fig. 3C, fig. S32, and table S7) (*9*, *11*, *49*, *54*, *56*–*73*). The certified PCE of the flexible PVSC is 25.55% (fig. S33) and the stabilized PCE is 25.4% after 300 s (fig. S34). External quantum efficiency (EQE) spectra (fig. S35) show excellent agreement between integrated *J*_sc_ and *J-V* measurements, further confirming the enhanced performance.

Light intensity–dependent *J-V* measurements (fig. S36, A and B) reveal linear *J*_sc_-light dependence across all devices, indicating suppressed bimolecular recombination (*74*). However, notable differences emerge in the *V*_oc_-light power law relationship. While mixed 3D devices show reduced slope (1.772 *k*T/*q*), indicative of partial trap passivation, the 2D/mixed 3D devices approach ideal behavior with a slope of 1.513 *k*T/*q*, demonstrating synergistic defect passivation achieved through MDA-doping and the interfacial 2D capping layer. These findings are corroborated by transient photovoltage and photocurrent decay measurements (fig. S36, C and D), where the 2D/mixed 3D device demonstrates the slowest voltage decay and the fastest current recovery, confirming efficient charge transport and suppressed recombination losses.

Long-term stability assessments reveal that unencapsulated flexible device based on 2D/mixed 3D perovskite maintains 97.8% initial PCE after 2000 hours of continuous operation under MPP tracking (ISOS-L-1 protocol) (*75*), markedly surpassing the performance of mixed 3D (87.0%) and control device (50.4%) (Fig. 3D) (*9*, *11*, *12*, *49*, *56*–*61*). This demonstrates a positive ability to retain efficiency and represents the highest stability among the reports (table S8). Accelerated aging tests under conditions of 85°C/85% RH (ISOS-D-3) demonstrate 89.9% PCE retention after 1000 hours for encapsulated 2D/mixed 3D device versus 50.5% for control device (Fig. 3E). Furthermore, thermal cycling from −40° to 85°C reveals 92.1% retention after 500 cycles for 2D/mixed 3D device, significantly higher than the 62.4% retention observed in control device (Fig. 3F), highlighting the exceptional environmental stability imparted by the strain homogenization strategies. To further evaluate performance under realistic day/night cycling, a condition known to induce recurrent lattice strain and accelerate degradation (*39*), devices have been subjected to periodic 12-hour light/12-hour dark cycles (fig. S37A). The 2D/mixed 3D device retains 97.2% of its initial PCE after 500 hours (∼21 cycles), markedly outperforming the mixed 3D (92.1%) and control (80.5%) devices (fig. S37, B to D). This result confirms that our strain-homogenization strategy effectively mitigates degradation from periodic thermal-expansion strain, a critical advancement for flexible PVSC operation. TOF-SIMS analysis after 500 hours at 85°C (fig. S38) shows reduced Ag^+^/Li^+^ distributions in 2D/mixed 3D device (*76*, *77*). This is corroborated by surface-sensitive XPS (fig. S39), which reveals a marked attenuated Ag 3d signal on the perovskite surface and I 3d signal beneath Ag layer of 2D/mixed 3D sample after aging, further confirming the suppression of both silver and iodine migration. Moreover, the 2D capping layer also increases the energy barrier for iodine ion migration, as confirmed by DFT calculations (figs. S40 and S41) (*78*). Together, these findings demonstrate that the 2D capping layer notably mitigates ion diffusion, contributing to the remarkable long-term stability of the devices by reducing lattice distortion and promoting a uniform distribution of thermal stress.

### Mechanical stability of flexible solar cells

**Fig. 4.**
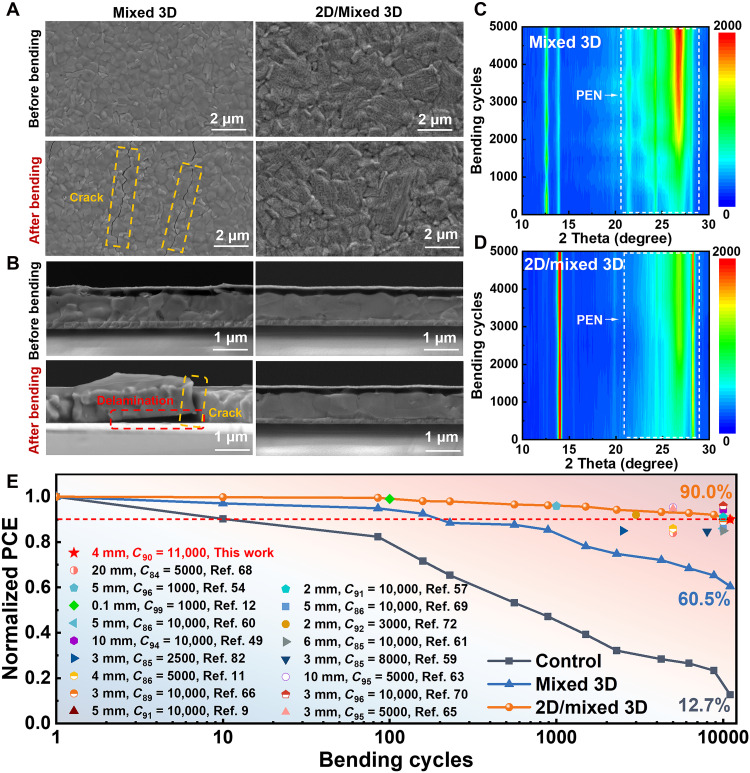
Mechanical stability of flexible PVSCs. (**A** and **B**) Top-view and cross-sectional SEM images of mixed 3D and 2D/mixed 3D perovskite films before and after bending 5000 cycles with a bending radius of 4 mm. (**C** and **D**) Semi–in situ XRD patterns of mixed 3D and 2D/mixed 3D perovskite films at different bending cycles with a bending radius of 4 mm. (**E**) Normalized PCE values for flexible PVSCs as a function of bending cycles with the bending radius of 4 mm and summary of bending stability of flexible PVSCs with different bending radius in recent years.

Mechanical stability constitutes a critical performance metric for the practical application of flexible PVSCs (*79*–*81*). We have systematically evaluated the mechanical durability of various device configurations through cyclic bending tests. Postbending SEM analyses reveal extensive cracking and delamination in mixed 3D device after 5000 cycles, whereas 2D/mixed 3D device maintain structural integrity (Fig. 4, A and B). This stark contrast underscores the critical role of the 2D layer in absorbing mechanical stress via its interwoven architecture and enhancing interfacial adhesion, thereby mitigating mechanical failure. The failure of mixed 3D device is primarily attributed to the accumulation of localized strain at grain boundaries and weak interfaces, which act as preferential sites for crack initiation and propagation under cyclic tensile stress (*7*). This process is quantified by their lower interfacial fracture energy (fig. S20). In contrast, the 2D/mixed 3D architecture addresses these failure pathways: The compliant 2D capping layer homogenizes the near-surface strain field and reinforces interfacial adhesion, effectively arresting cracks. Semi–in situ XRD analyses (Fig. 4, C and D) further corroborate the superior mechanical robustness of 2D/mixed 3D film, which exhibit no detectable PbI_2_ decomposition or substrate exposure peaks even after 5000 cycles, in stark contrast to the pronounced degradation observed in mixed 3D film.

As shown in fig. S42, after 1000 bending cycles at varying radii, 2D/mixed 3D device retains more than 97% of their initial PCE even at an extreme 4-mm bending radius, significantly outperforming mixed 3D (81%) and control device (49%). This exceptional mechanical flexibility is further validated through extended cyclic testing up to 11,000 cycles at a 4-mm radius (Fig. 4E), where 2D/mixed 3D devices retain 90% of their initial PCE, while control and mixed 3D devices deteriorate rapidly to 12.7 and 60.5%, respectively. Notably, the bending durability has been achieved in this work surpasses most of performances reported to date (table S9) (*9*, *11*, *12*, *49*, *54*, *57*, *59*–*61*, *63*, *65*, *66*, *68*–*70*, *72*, *82*), affirming the effectiveness of our strain homogenization strategy.

Finite element analysis offers additional mechanistic insights into the observed mechanical behavior (*83*, *84*). Simulations based on a 2D plane-strain model (fig. S43) reveal that the control device sustains a high average interfacial stress of 100.24 MPa, while the mixed 3D device shows a reduced value of 27.63 MPa. In contrast, the 2D/mixed 3D device exhibits a further lowered and homogenized stress distribution, with an average interfacial stress of 15.83 MPa. This progressive reduction correlates directly with the experimentally observed crack resistance and is attributed to the compliant 2D interlayer, which redistributes strain and mitigates local stress concentration, effectively delaying crack nucleation and propagation (schematically illustrated in fig. S44, with material properties summarized in table S10). Collectively, these results demonstrate that the incorporation of a 2D stress-release layer markedly enhances mechanical durability via dual mechanisms: (i) homogenizing strain distribution to prevent localized stress accumulation and (ii) reinforcing interfacial adhesion to inhibit crack initiation and delamination. Our findings highlight the pivotal role of strain engineering and interfacial design in advancing the mechanical robustness of flexible perovskite photovoltaics, providing both fundamental mechanistic insights and practical design guidelines for the realization of next-generation wearable and rollable solar technologies.

## DISCUSSION

In this work, we have systematically investigated A-site mixed-cation doping for lattice strain regulation and developed an interfacial engineering strategy that synergistically enhances both the mechanical robustness and optoelectronic performance of flexible PVSCs. Through rational cation alloying, we have significantly reduced defect densities and mitigated microstrain induced by compositional heterogeneity, thereby achieving homogeneous strain distribution across the perovskite films. To address residual PbI_2_ accumulation stemming from incomplete crystallization, we have introduced a diammonium spacer that in situ forms vertically aligned 2D perovskite layers. This interfacial modification not only effectively relaxes residual stress but also substantially improves charge transport and interfacial mechanical resilience. The optimized rigid and flexible PVSCs achieve a champion PCE of 26.59 and 25.88% (certified 25.55%, flexible), with unencapsulated devices retaining 97.8% of their initial PCE after 2000 hours of continuous MPP tracking under ISOS-L-1 protocols. Encapsulated devices exhibit exceptional long-term durability, maintaining 92.1% of their initial efficiency after 500 thermal cycles (−40° to 85°C) and 89.9% retention following 1000 hours of damp heat aging (85°C/85% RH). Notably, the synergistic effects of homogeneous strain regulation and enhanced interfacial toughness enable outstanding mechanical stability, with >90% efficiency retention after 11,000 bending cycles under extreme mechanical stress. These findings underscore the critical role of strain engineering and interfacial stress-release strategies in advancing both the performance and durability of flexible perovskite photovoltaics. Our dual approach of compositional optimization and interfacial design provides fundamental mechanistic insights and establishes a robust foundation for the practical realization of high-efficiency, mechanically resilient flexible perovskite photovoltaics, paving the way for their widespread application in portable electronics, wearable systems, and beyond.

## MATERIALS AND METHODS

### Material and reagent preparation

Ace (98%), GA (98%), MDA (98%), *N*,*N*-dimethylformamide (DMF; 99.8%), dimethyl sulfoxide (DMSO; 99.9%), acetonitrile (99.8%), chlorobenzene (CB; 99.8%), 2-propanol (IPA; 99.8%), and 4-tert-butyl pyridine (tBP; 98%) were purchased from Sigma-Aldrich. Tin(IV) oxide colloidal solution (SnO_2_; 15% in H_2_O) and lithium bis(trifluoromethylsulfonyl)imide (Li-TFSI; >98%) were purchased from Alfa Aesar. Formamidinium iodide (FAI; 99.8%), methylamine iodide (MAI; 99.5%), methylamine hydrochloride (MACl; 99.5%), octane diammonium iodide, EDOEA, and BDA were purchased from Xi’an Yuri Solar Co. Ltd. PbI_2_ (99.9985%), cesium iodide (99.99%), and 2,2′,7,7′-tetrakis [*N*,*N*-di(4-methoxyphenyl) amino]-9,9′-spirobifluorene (spiro-OMeTAD; 99%) were purchased from Advanced Election Technology Co. Ltd. ITO (transmission, >95%) substrates were purchased from South China Science & Technology Co. Ltd. Silver (Ag; 99.995%) was purchased from ZhongNuo Advanced Material (Beijing) Technology Co. Ltd. Unless specified, all chemicals were used as received without any further purification.

### Devices fabrication

Glass/ITO and polyethylene naphthalate (PEN)/ITO (Peccell, Japan) substrates were sequential ultrasonic cleaning in acetone, deionized (DI) water, and IPA for 20 min per step, followed by nitrogen drying and a 10-min air plasma treatment. For SnO_2_ deposition, a nanoparticle solution (15% SnO_2_ in H_2_O, diluted in DI water at a 1:3 ratio) was spin coated onto ITO substrates at 3000 rpm for 30 s and annealed at 150°C for 30 min. Flexible substrates were supported by a glass/polydimethylsiloxane platform to ensure uniform deposition. The PEN/ITO substrates were annealed at 120°C for 30 min. All substrates were subsequently treated with ultraviolet (UV) ozone for 10 min before transfer into a N_2_-filled glove box for further processing.

Perovskite films were fabricated using a conventional two-step deposition method (*85*, *86*). A 1.5 M PbI_2_ precursor solution in DMF and DMSO (9:1 v/v), was spin coated onto SnO_2_-coated substrates at 1500 rpm for 30 s, followed by annealing at 70°C for 1 min. For doped samples, Ace, GA, or MDA cations (1, 3, 5, or 10% molar ratios relative to PbI_2_) were introduced into the precursor solution. Organic salts (FAI/MAI/MACl in a 90-mg:6.9-mg:9-mg ratio dissolved in 1 ml of IPA) were spin coated onto PbI_2_ films at 2000 rpm for 30 s, followed by thermal annealing at 150°C for 15 min under ambient air (30 to 40% RH). For flexible devices, perovskite layers were annealed at 100°C for 1 min and then at 120°C for 15 min. Surface passivation was achieved by drop casting an EDOEA solution (5 mg/ml in IPA), spin coating at 5000 rpm for 30 s, and annealing at 100°C for 5 min. After cooling, a spiro-OMeTAD hole transport layer was deposited by spin coating at 4000 rpm for 30 s. The spiro-OMeTAD solution was prepared by dissolving 72.3 mg of spiro-OMeTAD in 1 ml of CB, with additives of 28.8 μl of tBP and 17.5 μl of Li-TFSI acetonitrile solution (520 mg/ml) with deep eutectic solvent (*87*). Last, a 100-nm Ag electrode was thermally evaporated through a shadow mask under a vacuum pressure of 7 × 10^−4^ Pa. All measurements were conducted at room temperature within a drying cabinet to ensure device stability.

### Module fabrication

First, ITO on PEN substrates was etched using a laser machine to form P1 lines. The substrates were cleaned sequentially with detergent, DI water, and ethanol under ultrasonication for 15 min each. SnO_2_ layer and perovskite layer were deposited via meniscus blade coating. After deposition of the Spiro-OMeTAD layer, the samples were reetched to form P2 lines, and the Ag was etched to form P3 lines, creating series-connected modules.

### Basic characterization

The current density–voltage (*J-V*) characteristics were measured using a Keithley 2400 source meter under simulated AM 1.5 G illumination at 100 mW cm^−2^ (Abet5 Sun2000 Solar Simulator). Calibration was performed with an National Renewable Energy Laboratory (NREL)-certified standard silicon solar cell. Forward scans ranged from 0 to 1.20 V, and reverse scans ranged from 1.20 to 0 V, with a step size of 20 mV and a scan rate of 0.2 V/s. Measurements were conducted in a N_2_ glove box to prevent environmental degradation. Morphological and structural analyses included SEM using an SU8020 microscope at an accelerating voltage of 5 kV and AFM using a Bruker MultiMode 8-HR system. High-resolution TEM was conducted on a JEOL JEM-F200 instrument. Crystallographic information was obtained by XRD using a Bruker D8 Discover 25 system. Chemical environments were probed using ^1^H nuclear magnetic resonance spectroscopy (BRUKER/AVANCE NEO 300 MHz). Optical properties were characterized using a Cary 5000 UV-Vis spectrophotometer (Agilent Technologies). Steady-state PL spectra were recorded on a Hitachi F-7000 spectrophotometer, and TRPL spectra were obtained using an Edinburgh Instruments FLS920 spectrometer. TA data were collected with a HARPIA spectroscopy system. The trap density of state was performed using Agilent 4294A. Surface and compositional analysis was performed via XPS using a Thermo Scientific ESCALAB 250Xi system. Electrical impedance spectroscopy was conducted using a Zahner electrochemical workstation across a frequency range of 1 to 10 MHz under 1-sun illumination at the open-circuit voltage. EQE was measured using an Oriel Cornerstone 260 1/4 m monochromator and calibrated with a monocrystalline silicon diode.

### Cross-sectional microstructure characterization

Cross-sectional TEM specimens were prepared using a dual-beam focused ion beam system (Helios G4, Thermo Fisher Scientific). A protective platinum layer was deposited via electron beam to safeguard the surface, followed by selective etching to extract lamella specimens. Lamellae were thinned to 150 to 200 nm using a gallium ion beam at 30 kV and 0.3 nA and then further reduced to 50 to 60 nm at 30 kV and 0.1 nA. To minimize ion implantation damage, final polishing was performed at 2 kV and 30 pA, with 15 to 20 s spent on each side. TEM imaging, including high-angle annular dark-field and high-resolution imaging, was carried out at 200 kV using a JEOL JEM-F200 instrument.

For cross-sectional measurements via PF-QNM and KPFM, devices were mechanically cleaved to expose cross sections. Simultaneous mapping of contact Young’s modulus, surface potential, and sample topography was conducted using a Bruker MultiMode 8-HR system.

Depth profiling of PVSCs was performed using a time-of-flight secondary ion mass spectrometer (PHI nanoTOF II) with a Bi^3+^ primary beam (30 keV, 2 nA) and an Ar sputter beam (3 kV, 100 nA). The sputtered area was 400 μm by 400 μm, and the sputter rate was 16 nm/min.

### Computational methods

Structural optimization was performed by Vienna Ab initio Simulation Package with the projector augmented wave method (*88*, *89*). The exchange functional was treated using the Perdew-Burke-Ernzerhof functional in combination with the DFT-D3 correction (*90*, *91*) to describe the weak interactions between atoms. The cutoff energy of the plane-wave basis was set at 450 eV in structural optimization. For the optimization of both geometry and lattice size, the Brillouin zone integration was performed with a Gamma *k*-point mesh of 0.04 Å^−1^. Partial occupancies of the Kohn-Sham orbitals were allowed using the Gaussian smearing method and a width of 0.05 eV. A geometry optimization was considered convergent when the energy change was smaller than 0.05 eV Å^−1^. Finite-element simulation of the devices was performed by ABAQUS finite element software to calculate the stress distribution of multilayer structures under bending.

### Stability measurement

Operational stability was evaluated by MPP tracking under continuous 1-sun white LED illumination. Initial *J-V* measurements were performed to determine the voltage at MPP. During stability testing, continuous illumination was applied except for brief intervals to calibrate the light source. Day/night cycling stability tests were carried out by alternating 12-hour periods of MPP tracking under 1-sun illumination (substrate temperature, ∼55°C) with 12-hour dark periods at room temperature (∼25°C). The devices requiring encapsulation were sealed using UV encapsulation adhesive (LT-U001, Luminescent Technology Corp.) and glass or PEN covered.

## Supplementary Materials

### This PDF file includes:

Notes S1 to S6 Figs. S1 to S44 Tables S1 to S10
