# Supplementary material for: Homogenizing out-of-plane strain distribution for high-performance flexible perovskite photovoltaics
Source: Sci Adv. 2026 Apr 10;12(15):eaec3238. doi: 10.1126/sciadv.aec3238 (PMC13068048; doi:10.1126/sciadv.aec3238)
Supplement: Supplementary file 1 — Notes S1 to S6 Figs. S1 to S44 Tables S1 to S10 [file sciadv.aec3238_sm.pdf]

Science Advances
AAAS

Supplementary Materials for

Homogenizing out-of-plane strain distribution for high-performance flexible perovskite photovoltaics

Yang Zhong *et al.*

Corresponding author: Licheng Tan, tanlicheng@ncu.edu.cn; Yiwang Chen, ywchen@ncu.edu.cn

Sci. Adv. **12**, eaec3238 (2026)
DOI: 10.1126/sciadv.aec3238

This PDF file includes:

Notes S1 to S6
Figs. S1 to S44
Tables S1 to S10

Supplementary Text

Note S1. Nanoindentation measurement.

Nanoindentation measurements were performed using a Bruker HYSITRON TI 980 system equipped with a Berkovich diamond indenter under ambient conditions. The experiments followed the standard Oliver-Pharr method. Nanoindentation measurements yield reduced modulus values, as described by the following equation:

$$\frac{1}{E_r} = \frac{1 - v_p^2}{E_p} + \frac{1 - v_i^2}{E_i} \tag{S1}$$

where E_p and v_p represent the Young's modulus and Poisson's ratio of the perovskite film, respectively, and E_i and v_i represent Young's modulus and Poisson ratio of the indenter (diamond), respectively.

Note S2. Williamson-Hall analysis.

We have analyzed the microstrain in the perovskite films through the Williamson-Hall (WH) equation:

$$\beta_T \cos\theta = \varepsilon(4\sin\theta) + \frac{K\lambda}{D} \tag{S2}$$

where B is the full width at half maximum (FWHM) of the perovskite peaks in the XRD patterns, θ is the diffraction angle, K is the shape factor, λ is the wavelength of the X-ray source and D is the crystallite size. The equation represents a linear relationship between $\beta_T \cos\theta$ and $4\sin\theta$, and the slope ε extracted here reveals the microstrain in the perovskite films.

Note S3. Calculations of the trap density.

At intermediate voltages, the trap-filled limit (TFL) regime was identified by a rapid nonlinear rise in the current where trap states were filled by the injected carriers. And the onset voltage (V_{TFL}) is proportional to the density of traps (n_t). The concentrations of trap states can be determined by equation:

$$V_{TFL} = \frac{e n_t L^2}{2\varepsilon\,\varepsilon^0} \tag{S3}$$

where e represents the elementary charge, L represents the film thickness, ε is the relative dielectric constant of perovskite, and ε_0 is the vacuum permittivity.

In addition, thermal admittance spectroscopy measurement has been carried out to evaluate the trap density of states (tDOS). The x-axis is converted from frequency to energy by equations: $E\omega = k_B T \ln\left(\frac{\omega_0}{\omega}\right)$, $\omega = 2\pi f$. The differentiated capacitance spectra at a specific temperature are superimposed to yield the energetic defect distribution:

$$N_{T(E\omega)} = -\frac{V_{b1}}{qW} \cdot \frac{dC}{d\omega} \cdot \frac{\omega}{k_B T} \tag{S4}$$

where q is the elementary charge, k_B is the Boltzmann's constant, T is the temperature, ω is the angular frequency, C is the capacitance.

Note S4. Macroscopic thermal stress.

The macroscopic thermal stress ($\sigma_{\Delta T}$) generated in a film on a substrate is described equation:

$$\sigma_{\Delta T} = \frac{E_p}{1 - V_p}\left(\alpha_s - \alpha_p\right)\Delta T \tag{S5}$$

where Ep is the Young's modulus of the perovskite film, ν_p is its Poisson's ratio (assumed to be 0.33), α_s and α_p are the coefficients of thermal expansion (CTE) of the substrate and the film, respectively, and ΔT is the temperature change relative to the stress-free temperature.

Note S5. Double cantilever beam (DCB) fracture toughness measurement.

Double cantilever beam (DCB) specimens were prepared in a symmetric "sandwich" configuration to measure the cohesive fracture energy within the perovskite composite stack. The layer structure is as follows (see schematic in **Fig. S35A**): PEN/ITO/SnO_2/Perovskite/UV adhesive /PEN. Tests were performed under displacement control (2 mm min^{-1}) using a universal testing machine (MTS-E43.104).

The crack length (a) during stable propagation was determined from the unloading compliance using:

$$a = \left(\frac{d\Delta}{dP} \times \frac{BEh^3}{8}\right)^{\frac{1}{3}} - 0.64h \qquad (S6)$$

where B is the width, E is the Young's modulus of the substrate, and h is the half-thickness of the DCB specimen.

The fracture energy (G_c) was calculated as:

$$G_c = \frac{12P_C^2a^2}{B^2Eh^3}\left(1 + 0.64\frac{h}{a}\right)^2 \qquad (S7)$$

where P_C is the critical load at crack initiation.

The load-displacement curves (**Fig. S35B**) show a ~44% higher critical load for the 2D/mixed 3D film (59.95 N) compared to the mixed 3D film (41.73 N). The calculated fracture energy (**Fig. S35C**) increases from 0.55 J m^{-2} (mixed 3D) to 1.71 J m^{-2} (2D/mixed 3D).

Note S6. GIXRD Residual strain gradient measurement.

This analysis focuses on quantifying the macroscopic residual strain gradient (often termed macrostrain) across the film thickness. This is a distinct physical quantity from the local microstrain (quantified via Williamson-Hall analysis in Note S2), which arises from lattice imperfections within individual grains. The $\sin^2\psi$ method employed here measures coherent variations in lattice spacing over long ranges, reflecting film-level stresses induced by processing, thermal mismatch, or interfacial constraints.

X-ray diffraction (XRD) represents a common technique to evaluate residual stress/strain gradients along the surface normal direction in polycrystalline thin films and coatings from measured X-ray elastic strains using the $\sin^2\psi$ technique. The measured lattice spacing d and X-ray elastic strains ε represent volume-average quantities which depend on the actual stress/strain depth profile, X-ray penetration depth, reflection plane (hkl) and experiment geometry. In general, d and ε can be related by $\varepsilon = (d-d_0)/d_0$. According to the Braggs law, Hooke's law and equations of equilibrium, the classic $\sin^2\psi$ equation about stress σ and 2θ can be obtained as follow:

$$\sigma = -\frac{E}{2(1+\nu)} \cdot \frac{\pi}{180} \cdot cot\theta_0 \frac{\partial(2\theta)}{\partial sin^2\psi} \qquad (S8)$$

E and υ are Young's modulus and Poisson's ratio of the thin film, respectively. θ_0 is the diffraction peak for stress free perovskite (hkl) crystal plane and θ is the diffraction peak for the actual perovskite films. The ψ is the angle the diffraction vector with respect to the sample normal direction.

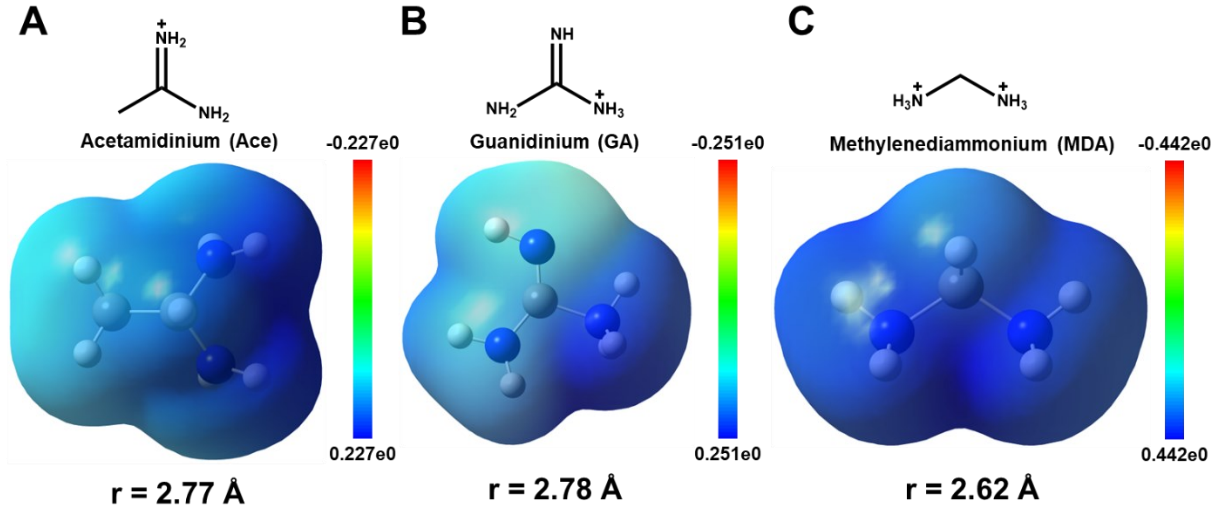

Fig. S1. Chemical structures and electrostatic potential distributions of different A-site cations.

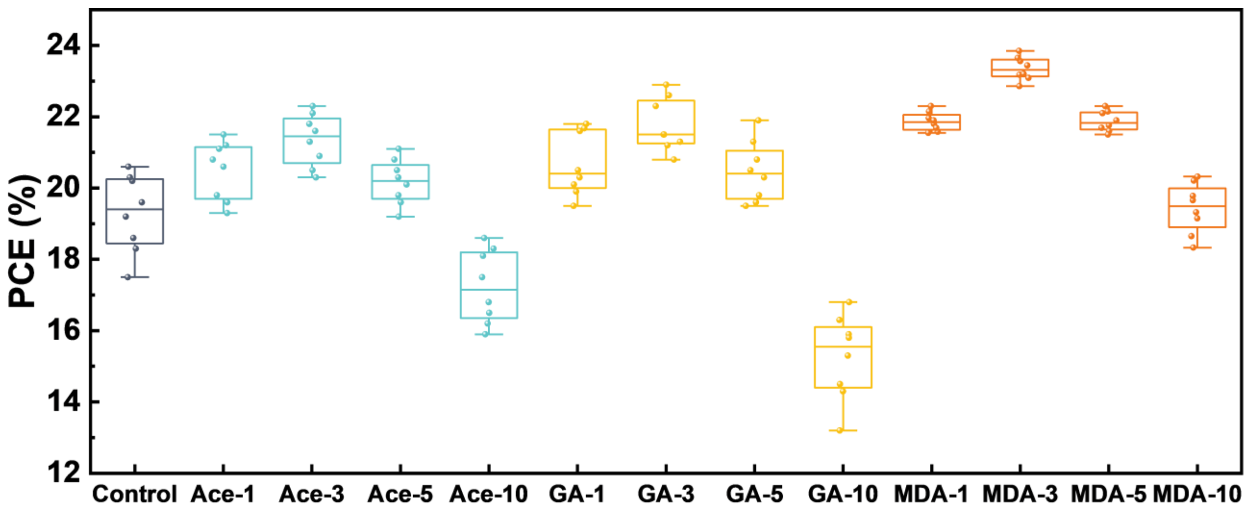

Fig. S2. PCE distribution of flexible PVSCs fabricated with varying mixing ratios of different A-site cations.

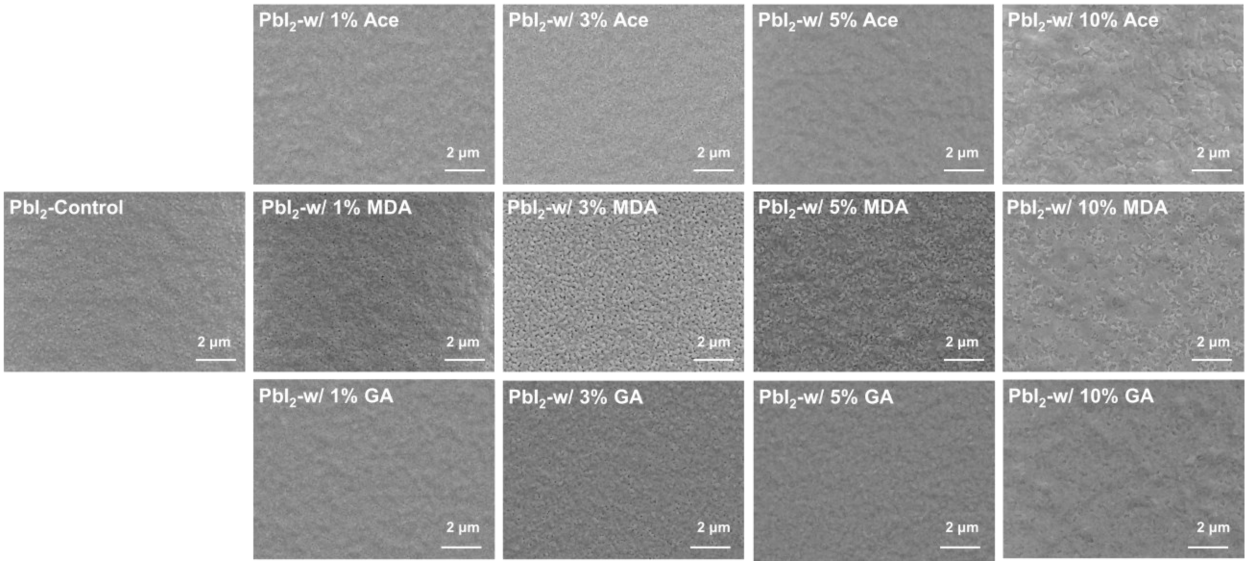

Fig. S3. Top-view SEM images of PbI_2 films with varying mixing ratios of different A-site cations. MDA^{2+}-modified PbI_2 films develop a highly porous morphology that facilitates uniform penetration of organic cations during sequential deposition, promoting homogeneous nucleation and growth. In contrast, Ace^+- and GA^+-incorporated PbI_2 films exhibit compact structures that likely restrict cation diffusion, leading to heterogeneous crystallization.

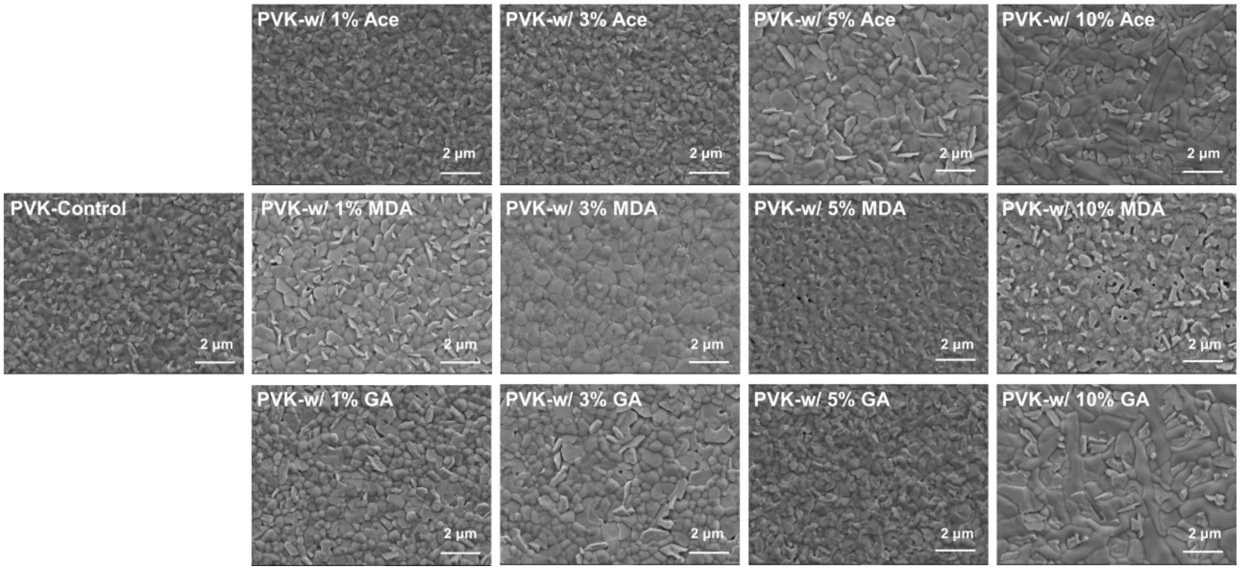

Fig. S4. Top-view SEM images of perovskite films with varying mixing ratios of different A-site cations.

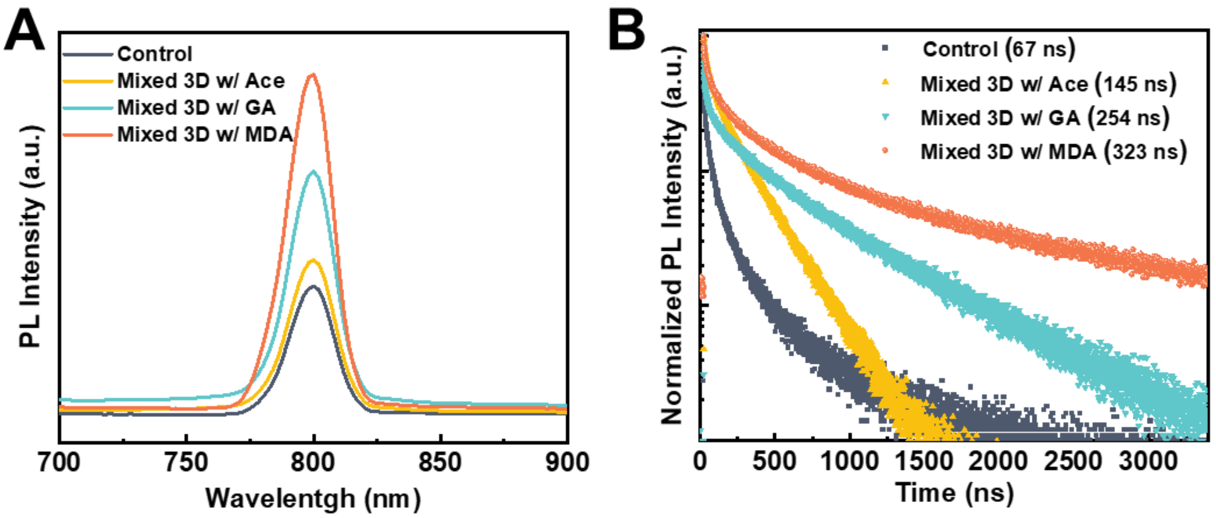

Fig. S5. PL and TRPL spectra of perovskite film with different A-site cation mixtures. MDA^{2+}-doped films demonstrate enhanced PL intensity and prolonged carrier lifetime, indicative of superior charge transport property and reduced trap density.

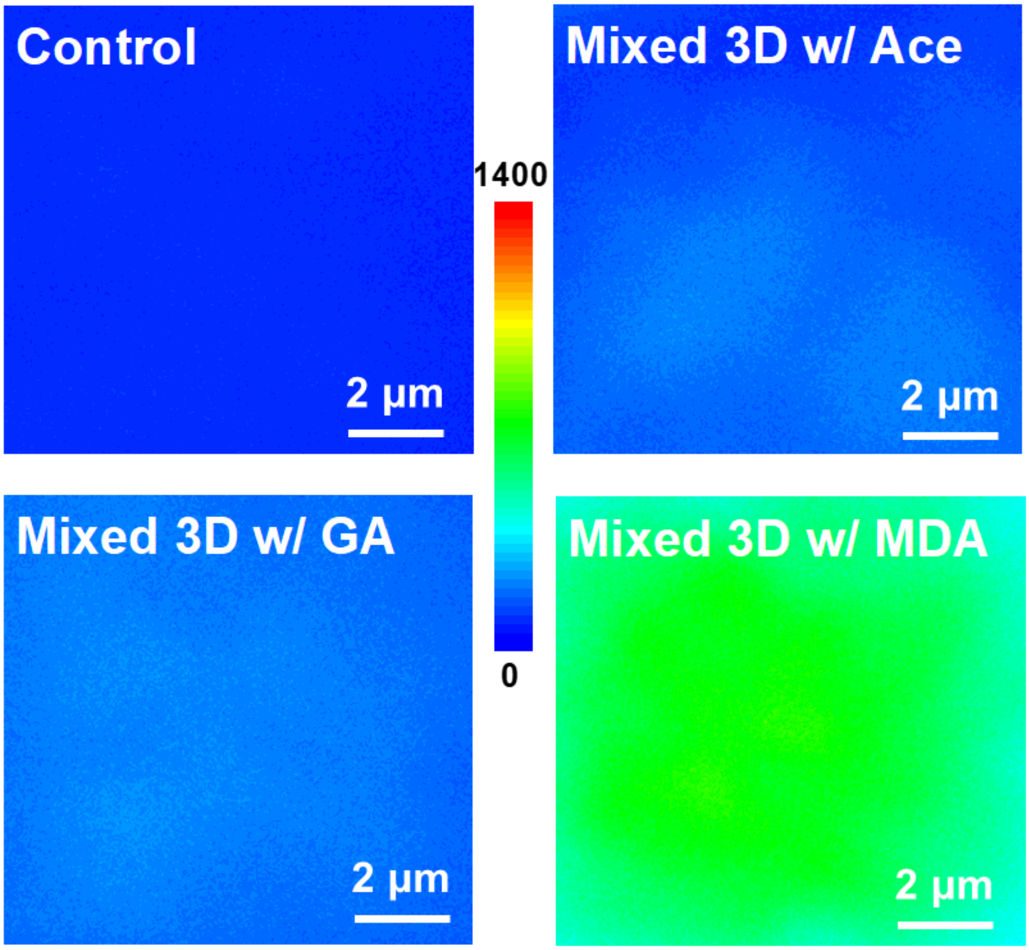

Fig. S6. PL mapping images of perovskite films with different A-site cation mixtures.

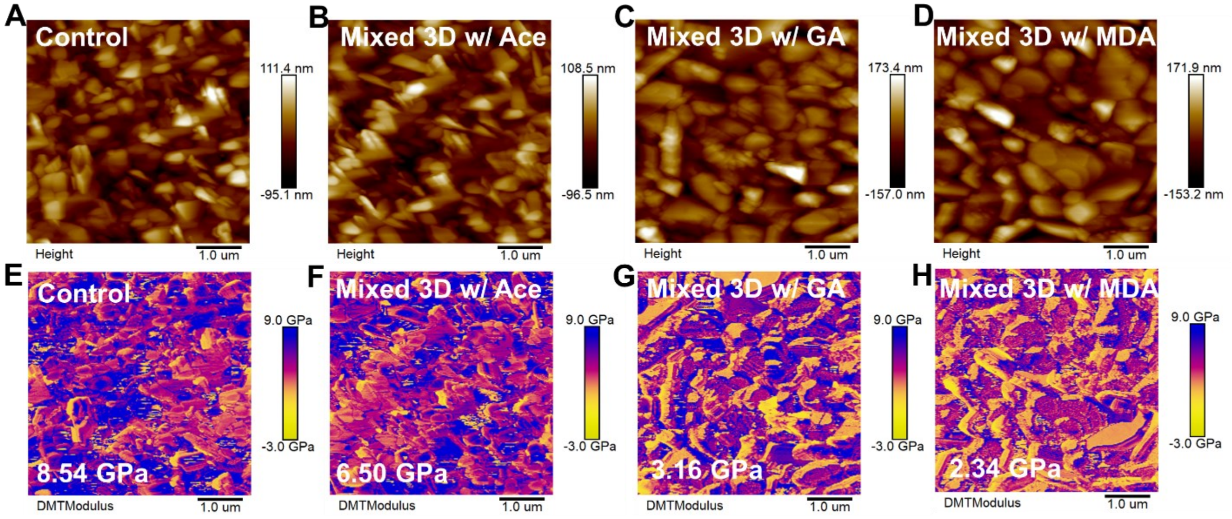

Fig. S7. AFM topography images and corresponding surface PF-QNM modulus measurement of perovskite films with different A-site cation mixtures.

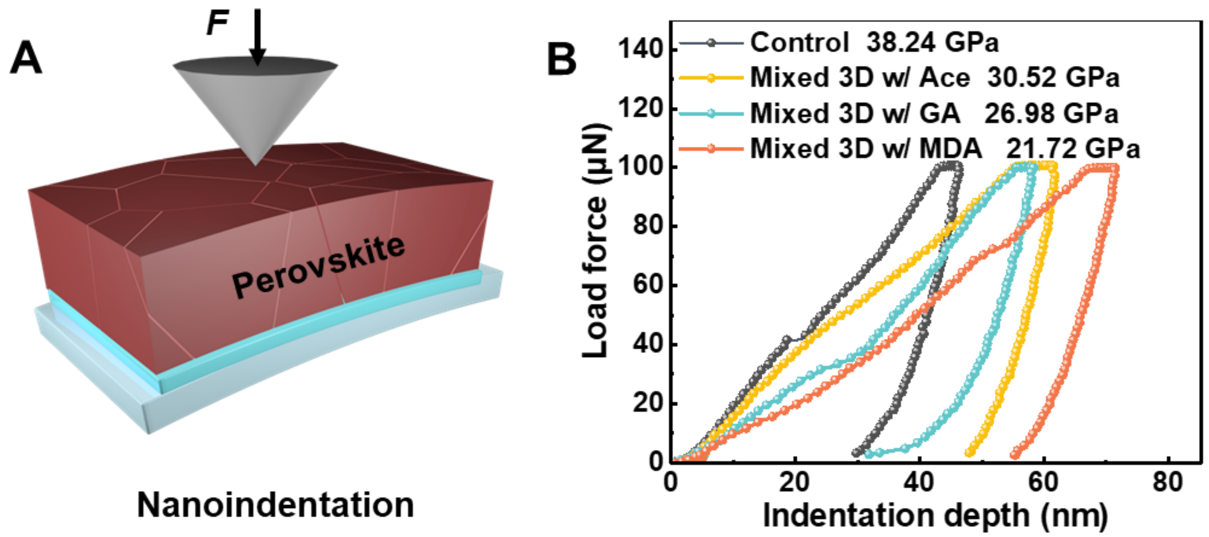

Fig. S8. Nanoindentation analysis of perovskite films. (**A**) Schematic diagram of the nanoindentation measurements. *F*, force. (**B**) Load force-dependent indentation depth curve of perovskite film with different A-site cation mixtures.

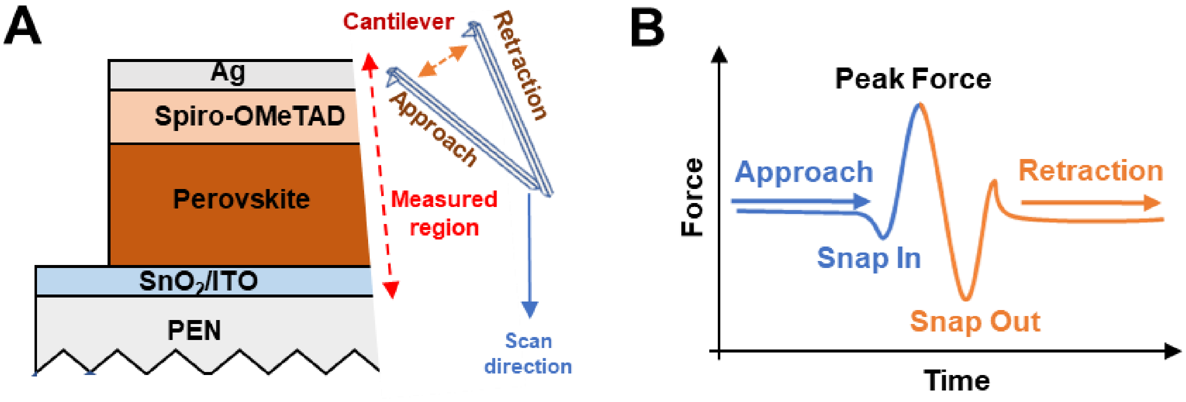

Fig. S9. PF-QNM modulus measurement principle and setup. (**A**) Schematic illustrating the PF-QNM modulus measurement setup and planar device structure of ITO/SnO_2/perovskite/spiro-OMeTAD/Ag. (**B**) Schematic of the force-distance curve obtained at each tapping cycle with the peak force as the controlling parameter.

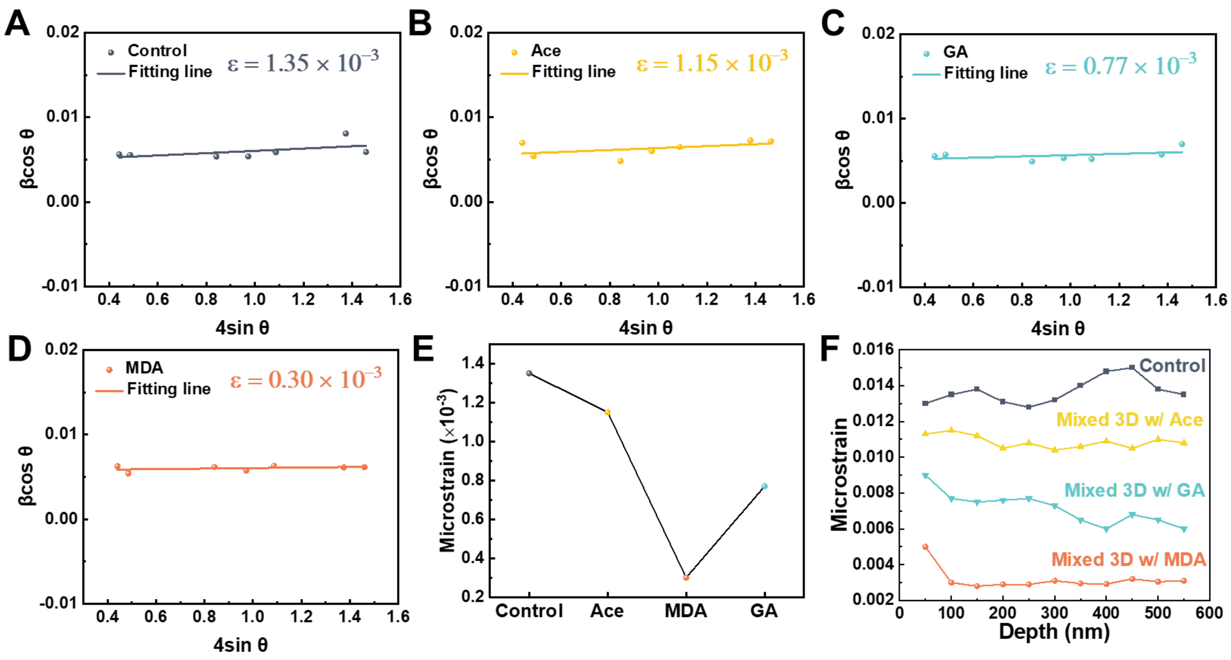

Fig. S10. Williamson-Hall analysis of perovskite films. (**A** to **D)** Williamson-Hall plots, (**E**) microstrain and (**F**) corresponding depth profiles of perovskite films with different A-site cation mixtures.

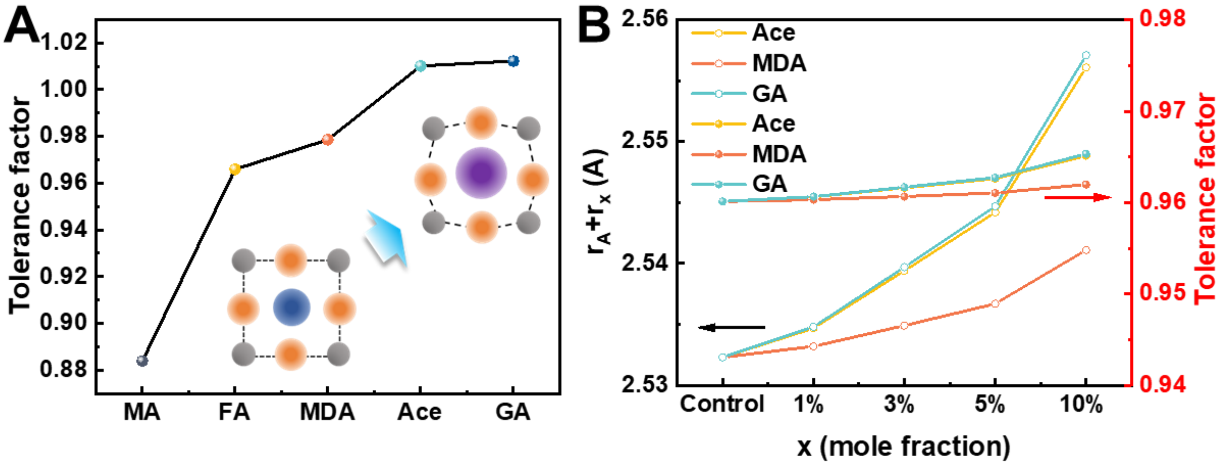

Fig. S11. Effect of A-site cations on the Goldschmidt tolerance factor of perovskites. (**A**) Goldschmidt tolerance factor of $APbI_3$ perovskites with different A-site cations. (**B**) Tolerance factors calculated with different x values in $FA_{0.93}MA_{0.07-x}A_xPbI_3$. A is the cations including Ace, MDA and GA.

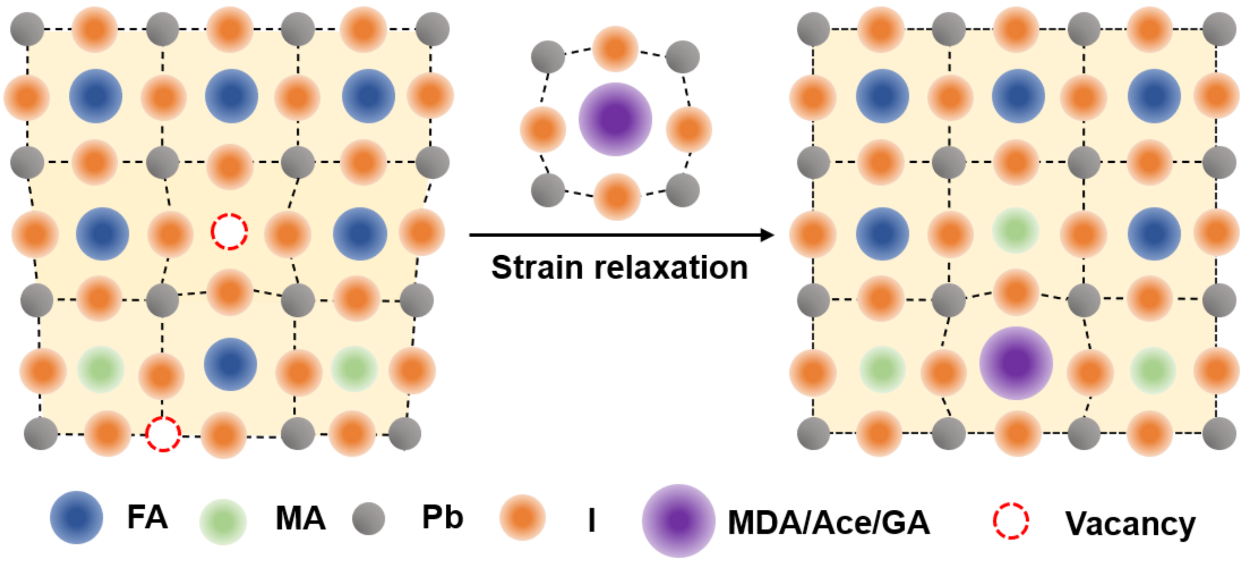

Fig. S12. Strain relaxation mechanism by mixed A-site cations. Schematic illustration of the proposed distribution by incorporating different A-site cations within the perovskite crystal for compensating the lattice strain induced by difference between the radius of A-site cations.

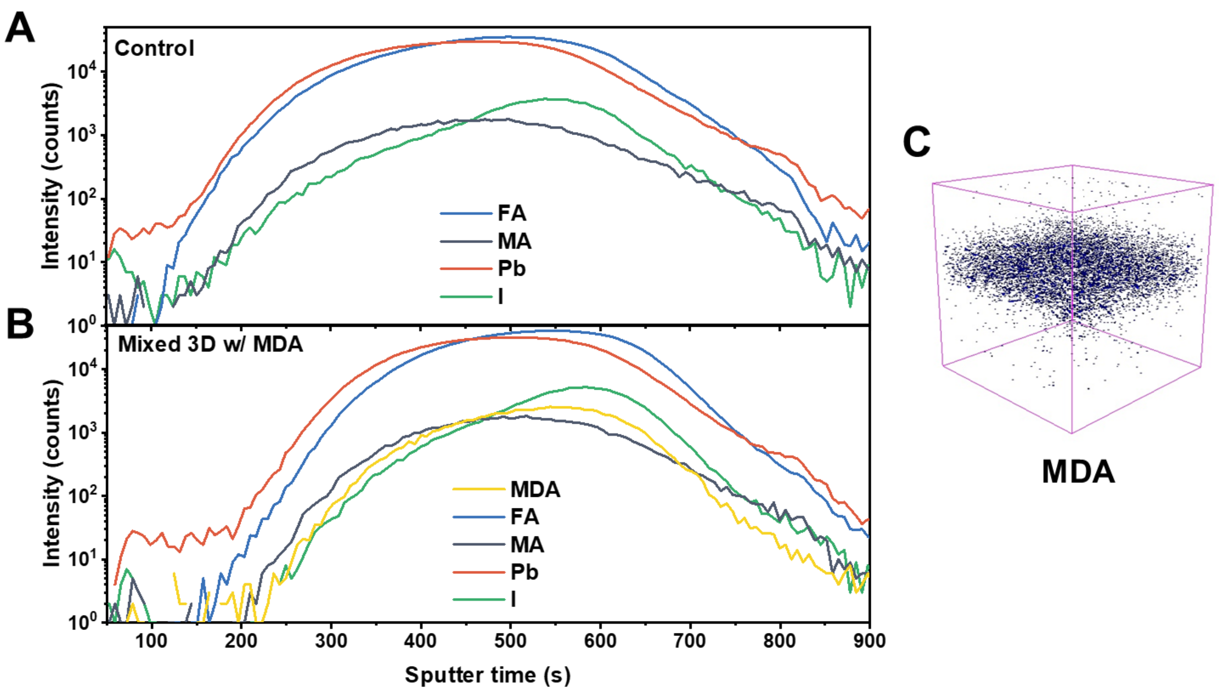

Fig. S13. Distribution of cations obtained from TOF-SIMS spectra for control and MDA-doped perovskite.

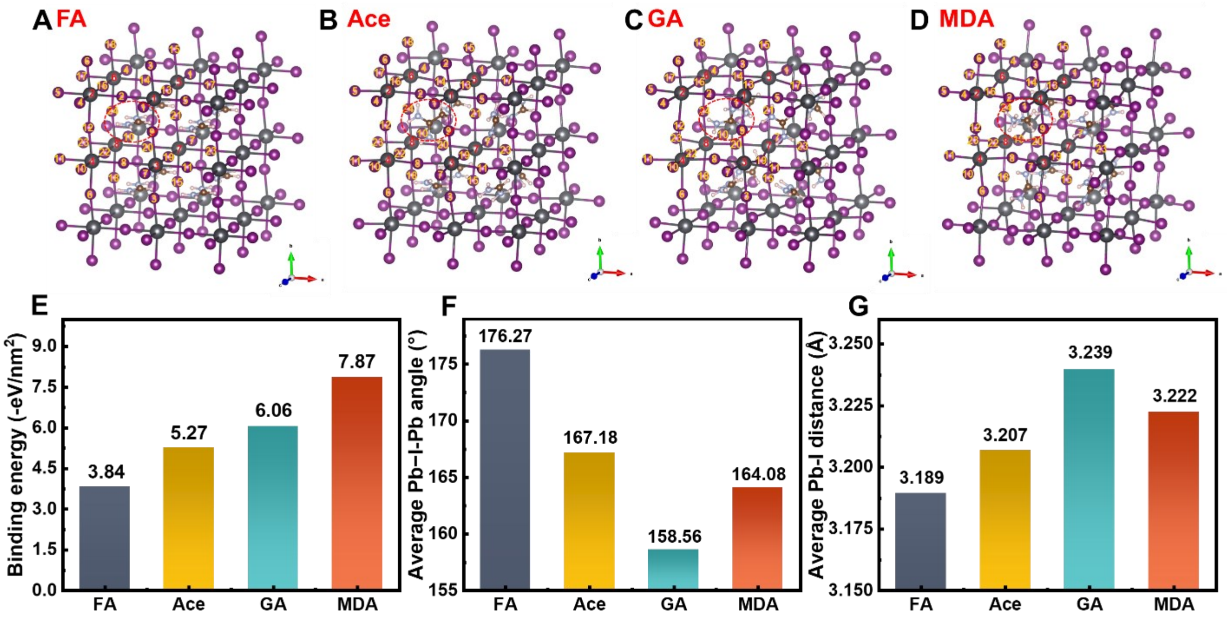

Fig. S14. Theoretical modeling and calculation results of perovskite systems with different A-site cation mixtures. (**A** to **D**) Theoretical models and (**E** to **G**) calculation results of perovskite systems with different A-site cation mixtures.

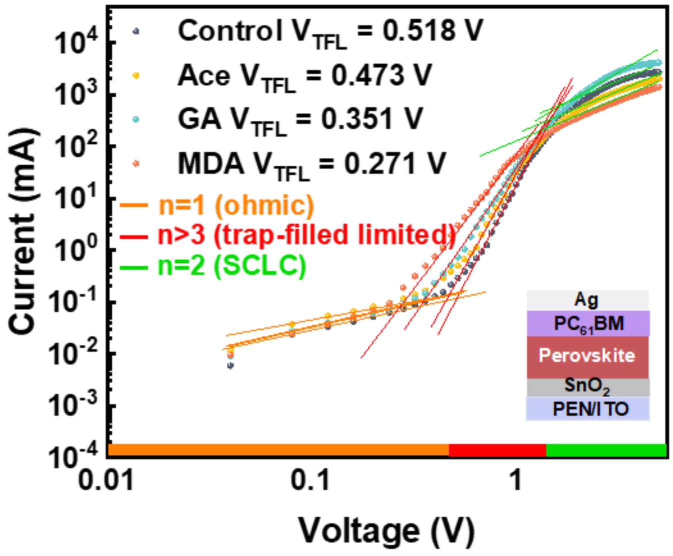

Fig. S15. SCLC measurement of electron-only perovskite devices. *J-V* curves for the electron-only devices with structure of ITO/SnO_2/perovskite/$PC_{61}BM$/Ag based on SCLC model.

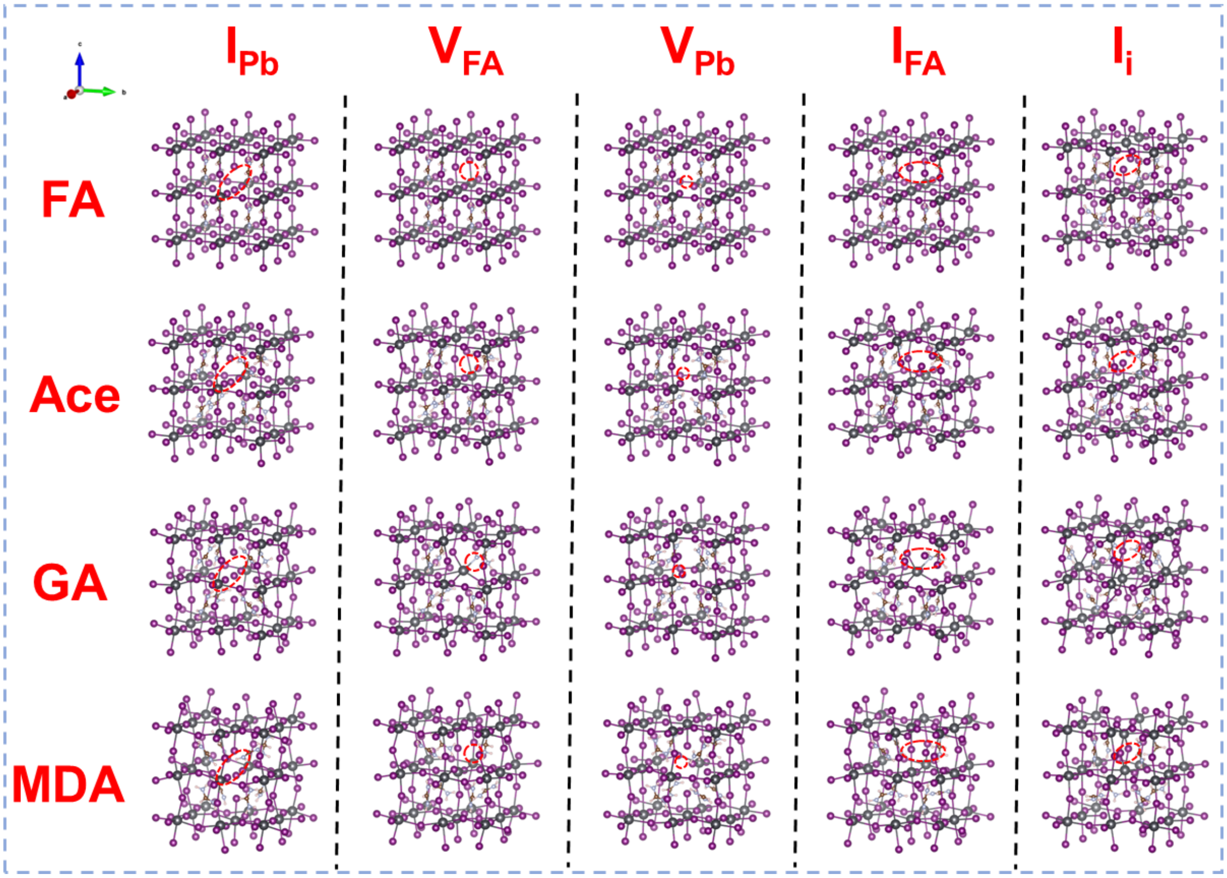

Fig. S16. Schematic of defect types in $FAPbI_3$ with and without cation incorporation. Graphical illustrations of $FAPbI_3$ perovskite systems with or without cation incorporation with the negatively charged defects, including iodine-lead anti-sites (I_{Pb}), formamidinium vacancies (V_{FA}), lead vacancies (V_{Pb}), iodine-formamidinium anti-sites (I_{FA}) and iodine interstitials (I_i).

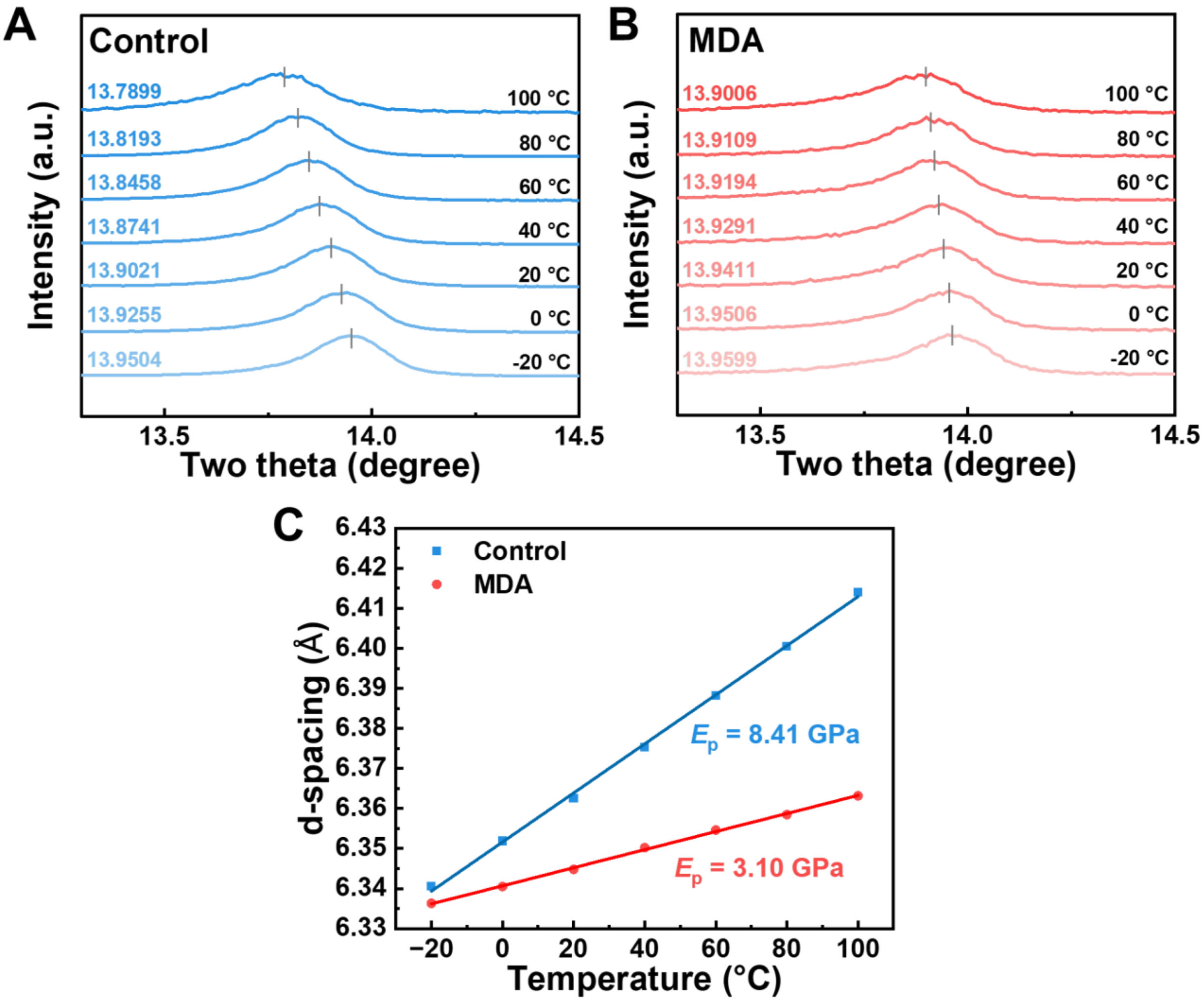

Fig. S17. Temperature-dependent X-ray diffraction analysis. (100) diffraction peaks for the (**A**) control and (**B**) MDA-doped mixed-cation perovskite films measured across a temperature range of -20 °C to 100 °C. (**C**) Temperature-dependent (100) d-spacing of control and MDA-doped mixed-cation perovskite films.

The control film exhibits a pronounced shift in peak position from 13.9504° to 13.7899°, indicating substantial thermal lattice strain. In contrast, the MDA-doped film shows a markedly smaller shift (from 13.9599° to 13.9006°), demonstrating enhanced thermal lattice stability. The Young's moduli (*E*) derived from the thermal strain data are 8.41 GPa for the control and 3.10 GPa for the MDA-doped film.

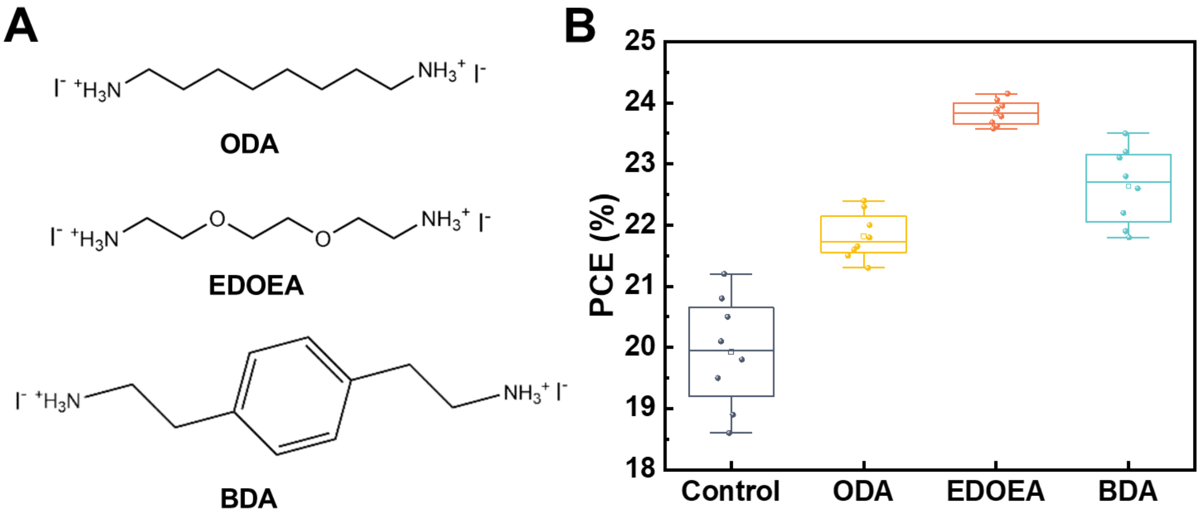

Fig. S18. Molecular structures and photovoltaic performance of flexible PVSCs with various cations. (**A**) Molecular structure of ODA, EDOEA and BDA. (**B**) PCE distribution of flexible PVSCs based on 2D/mixed 3D perovskite films with various cations.

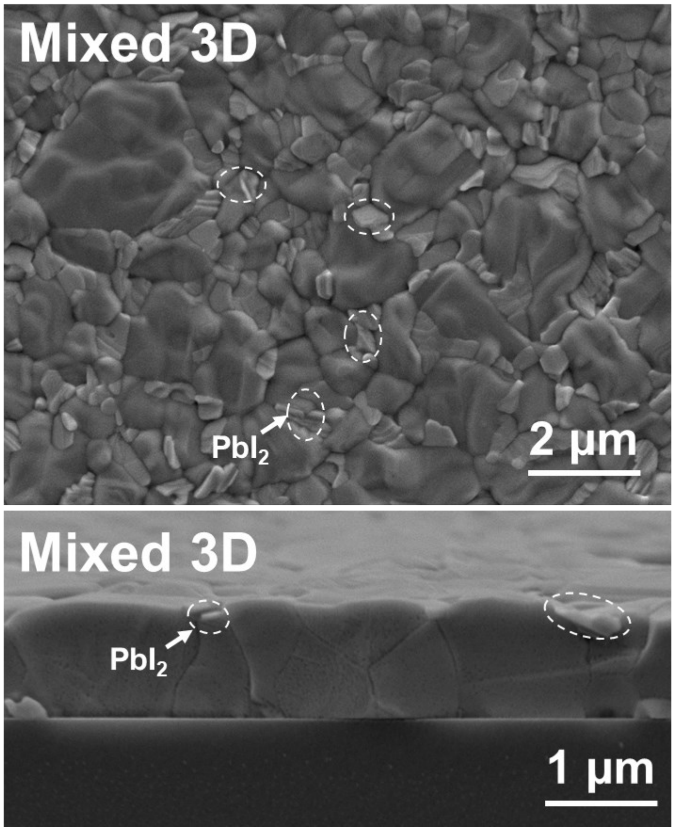

Fig. S19. Top-view and cross-section SEM images of mixed 3D perovskite film.

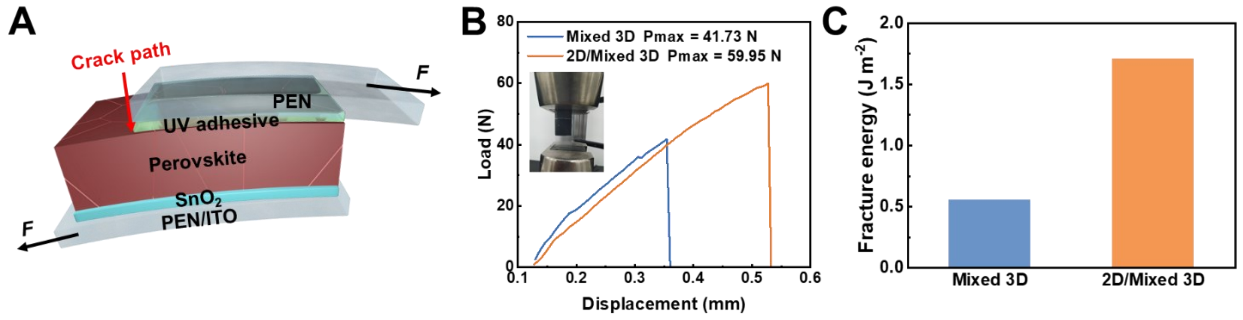

Fig. S20. Fracture energy measurement of perovskite films by double cantilever beam test. (**A**) Schematic illustration of double cantilever beam (DCB) test. (**B**) Load-displacement curves for the films for the measurement of G_c of the "sandwich" DCB specimens. (**C**) Fracture energy of the mixed 3D and 2D/mixed 3D perovskite films.

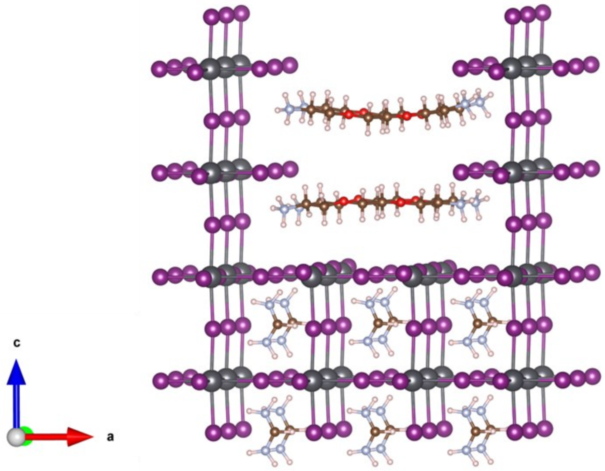

Fig. S21. Theoretical model of the vertically oriented DJ-phase 2D perovskite grows epitaxially on the 3D surface.

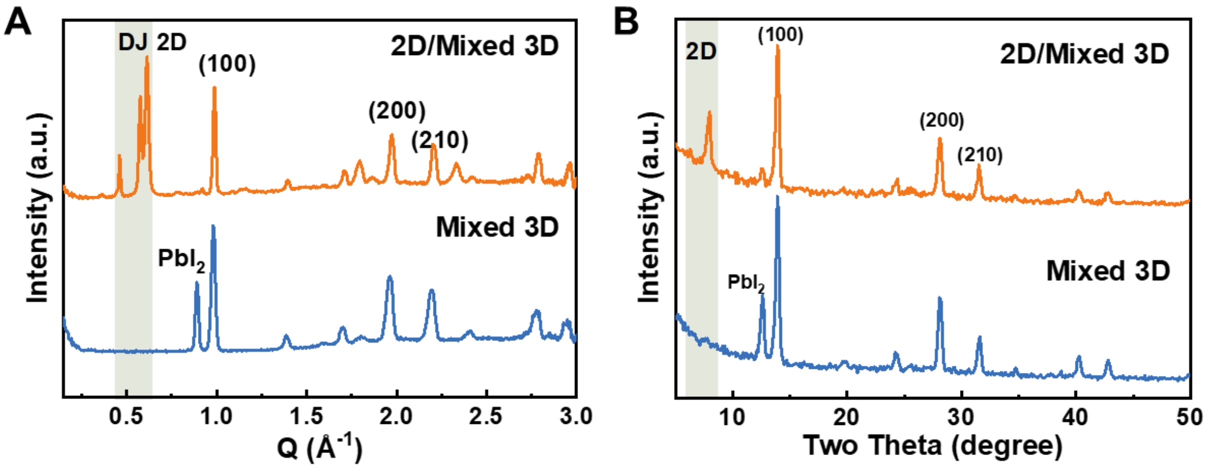

Fig. S22. Structural characterization of perovskite films by GIWAXS and GIXRD. (**A**) The corresponding intensity profiles as a function of the scattering vector Q from GIWAXS patterns and (**B**) GIXRD pattern of mixed 3D and 2D/mixed 3D perovskite films.

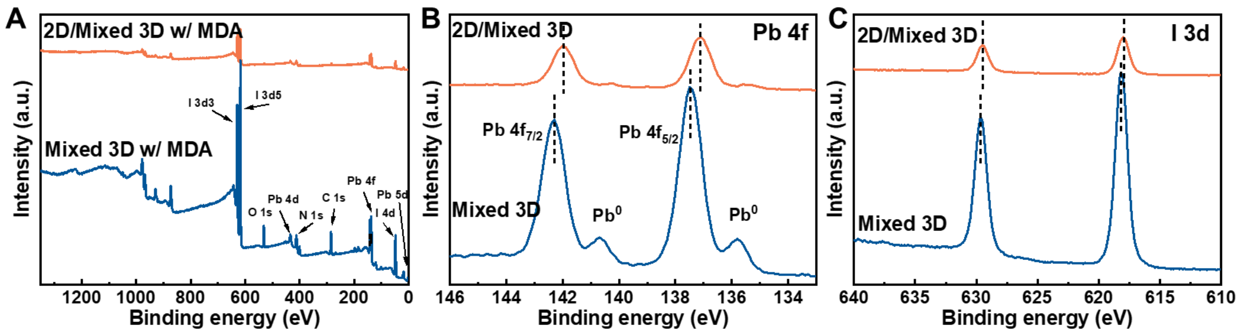

Fig. S23. XPS characterization. XPS spectra of full scan, Pb 4f and I 3d for mixed 3D and 2D/mixed 3D perovskite films.

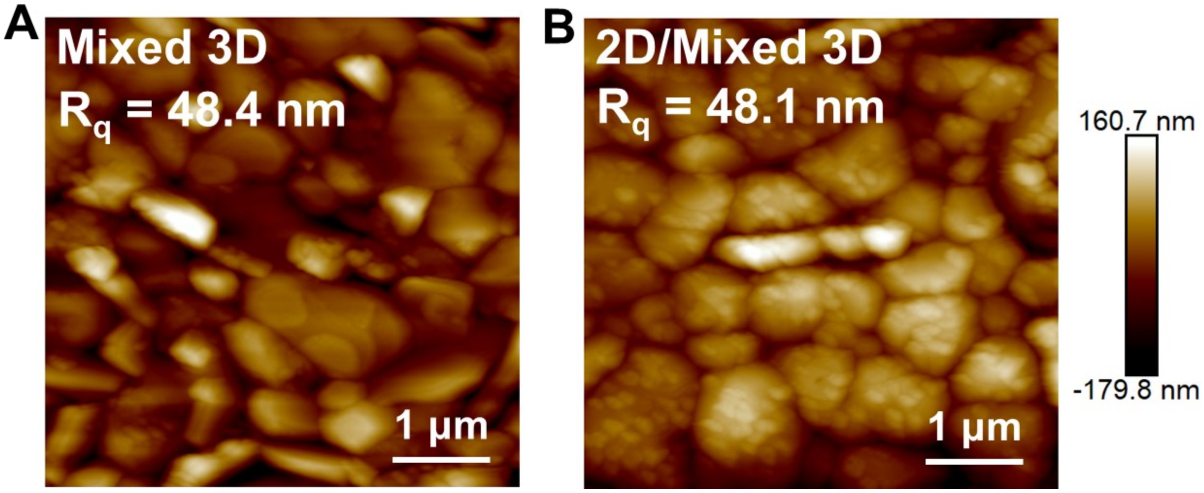

Fig. S24. Surface AFM measurement of mixed 3D and 2D/mixed 3D perovskite films.

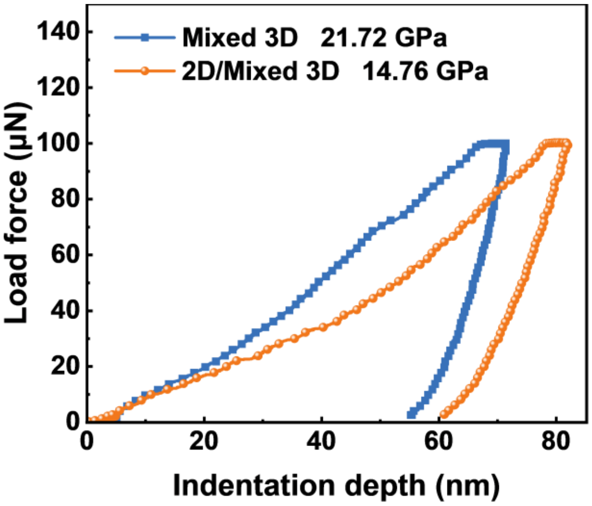

Fig. S25. Load force-dependent indentation depth curve of mixed 3D and 2D/mixed 3D perovskite films.

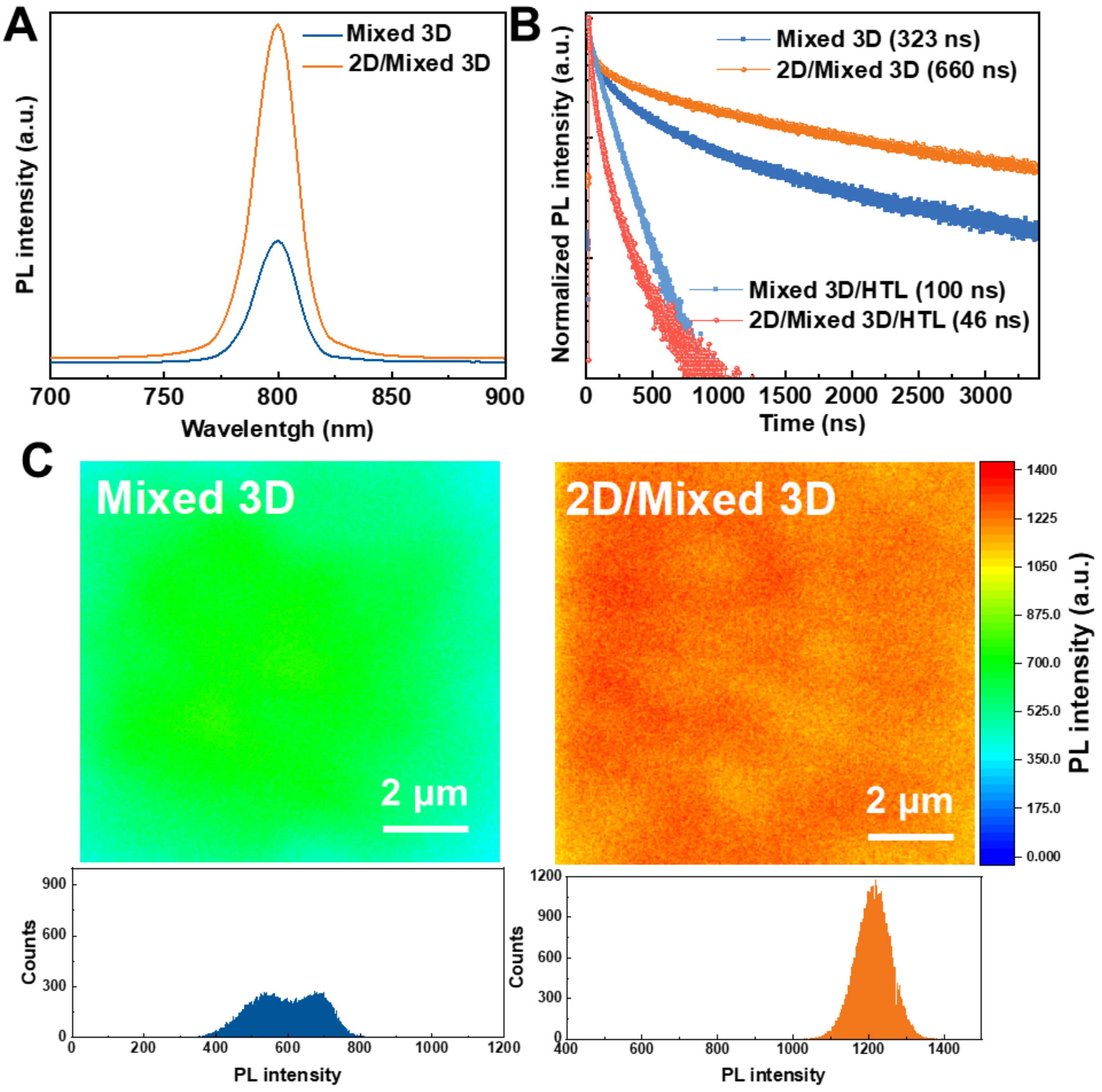

Fig. S26. Steady-state and time-resolved PL analysis. (**A**) PL, (**B**) TRPL and (**C**) PL mapping and distributions of mixed 3D and 2D/mixed 3D perovskite films.

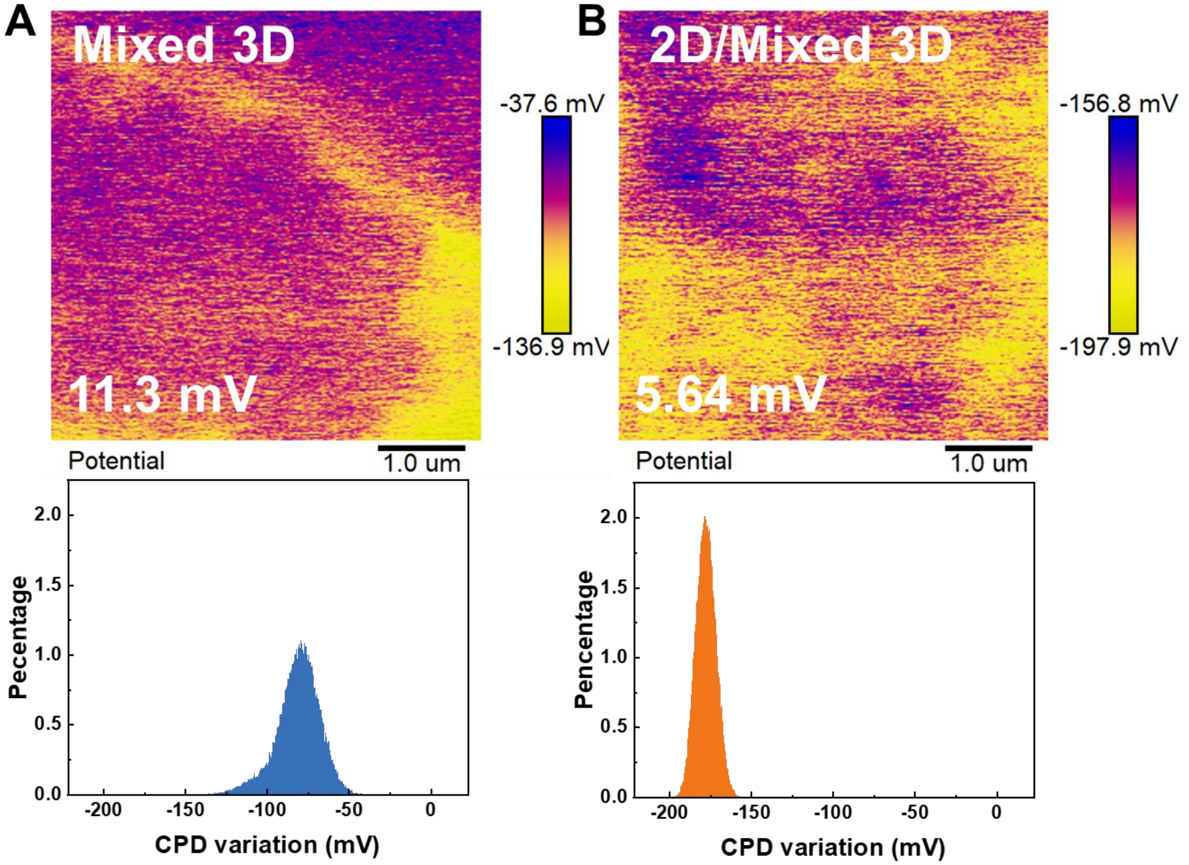

Fig. S27. KPFM surface potential maps and statistics diagrams of mixed 3D and 2D/mixed 3D perovskite films.

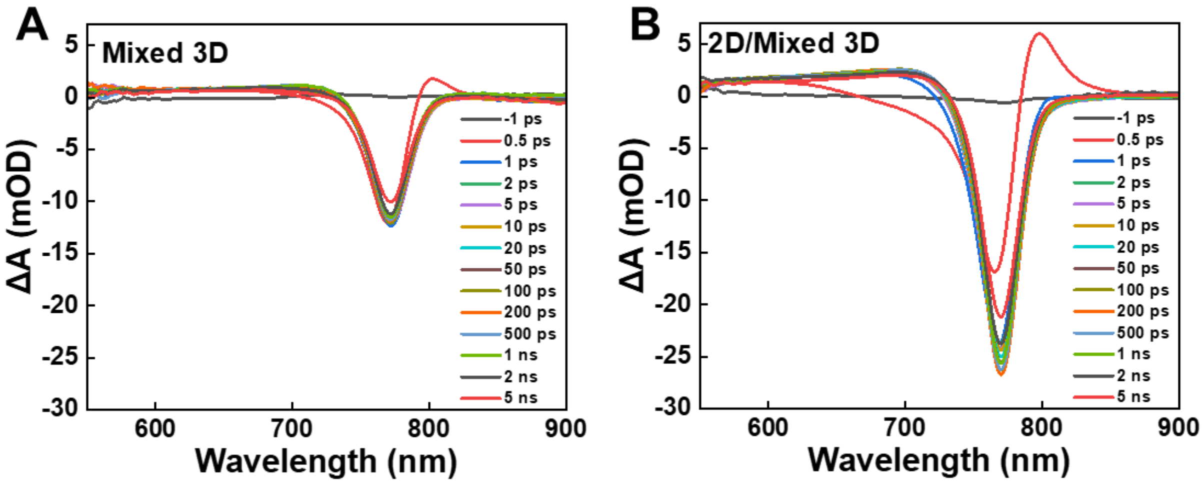

Fig. S28. Pseudocolor TA spectrum plot of mixed 3D and 2D/mixed 3D perovskite films.

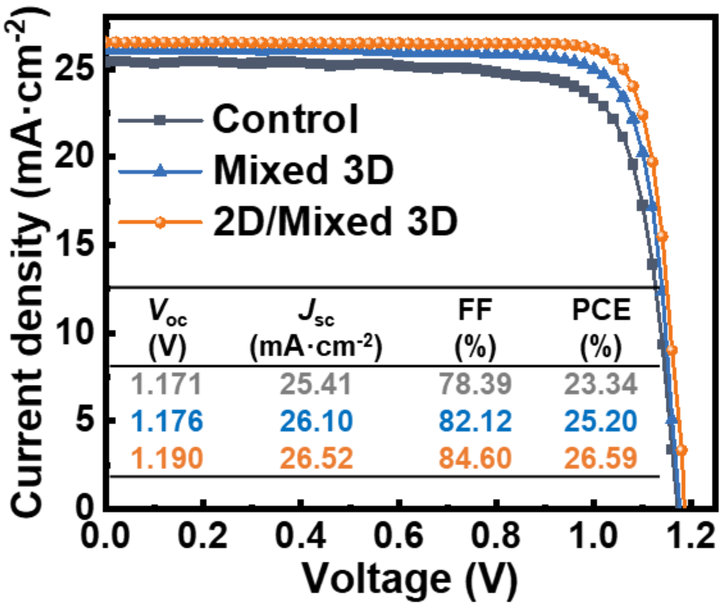

Fig. S29. ***J-V*** **curves of champion rigid PVSCs based on control, mixed 3D and 2D/mixed 3D perovskite films.**

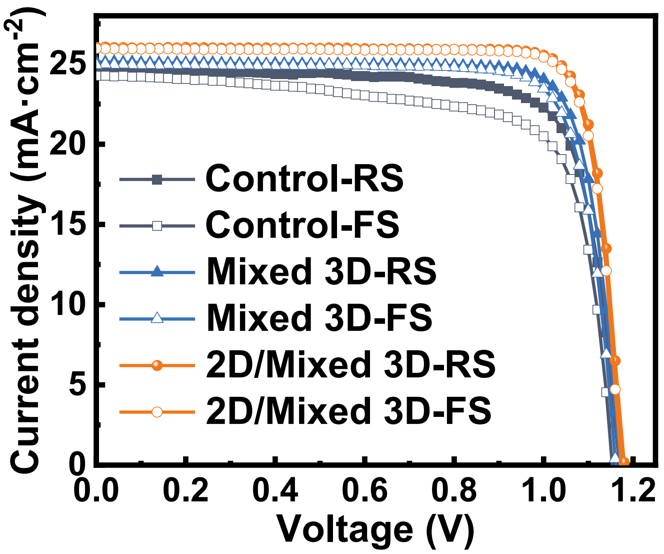

Fig. S30. The reverse and forward scanned *J-V* curves of champion flexible PVSCs based on control, mixed 3D and 2D/mixed 3D perovskite films.

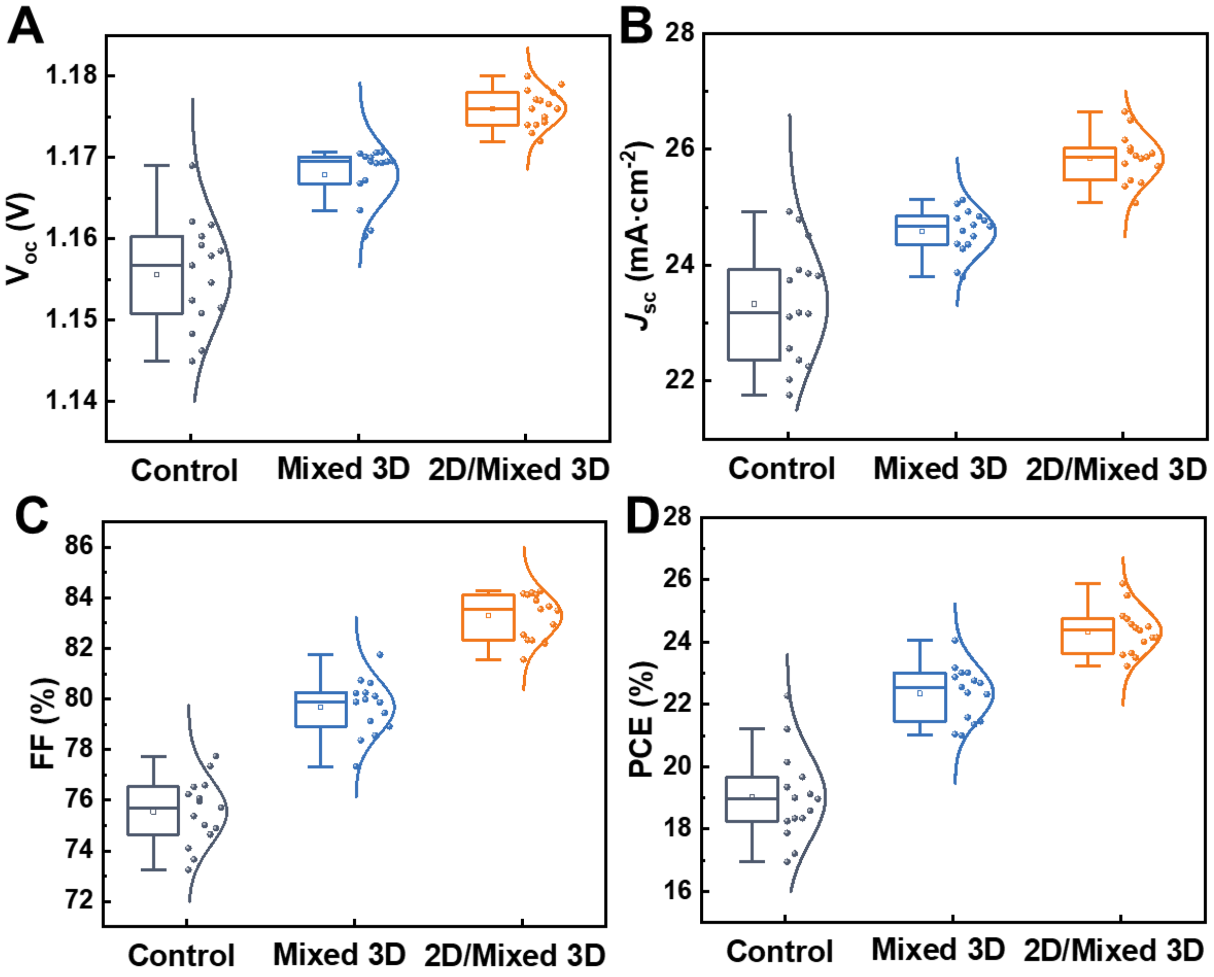

Fig. S31. Photovoltaic parameter distribution of flexible PVSCs based on control, mixed 3D and 2D/mixed 3D perovskite films.

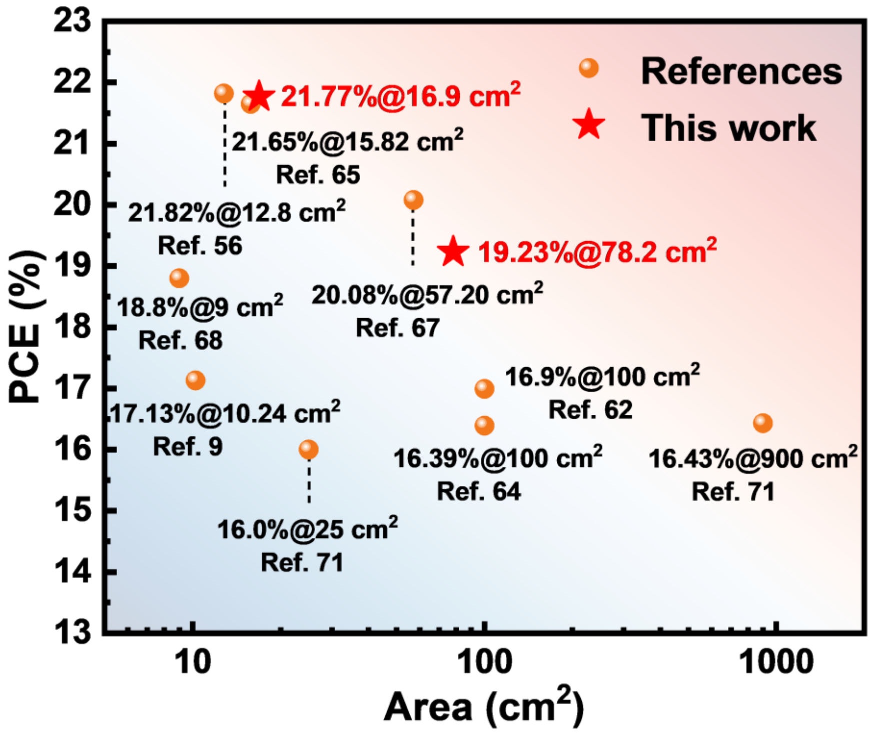

Fig. S32. Statistical diagram of PCE obtained from recently reported flexible PVSMs.

华南国家计量测试中心
广东省计量科学研究院
SOUTH CHINA NATIONAL CENTER OF METROLOGY
GUANGDONG INSTITUTE OF METROLOGY

校 准 结 果
RESULTS OF CALIBRATION

证书编号 NYX202500021 Certificate No. | 原始记录号 NYX202500021 Record No. | 第 4 页,共 5 页 Page of

1、外观检查： 符合要求
Apparent Inspection: Pass.

2、测试条件： 温度(25±2)℃；辐照度1000W/m^2。
Test conditions: Temperature: (25±2)℃；Irradiance：1000W/m^2.

3、电流-电压特性曲线和功率-电压特性曲线：
I-V and P-V curves:

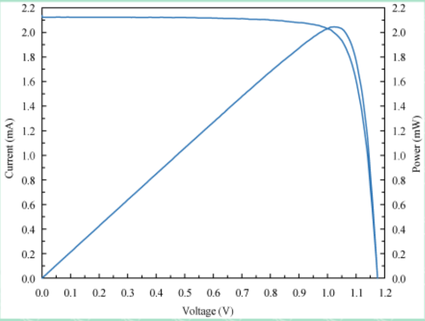

图1 电流-电压特性曲线和功率-电压特性曲线
Figure1 I-V and P-V characteristic curves

4、光电性能参数：
Results of photoelectric properties:

表1(Table 1)

面积	短路电流密度J_{sc}	短路电流 I_{sc}	开路电压 V_{oc}	填充因子 FF	最大功率 P_m	最佳工作电流I_m	最佳工作电压V_m	转换效率 η
Area	Short ciruit current density	Short ciruit current	Open circuit voltage	Fill factor	Maximum power	Optimum working current	Optimum working voltage	Efficiency
cm^2	mA/cm^2	mA	V	%	mW	mA	V	%
0.0800	26.55	2.124	1.173	82.08	2.044	2.004	1.020	25.55

Fig. S33. Certification measurement report of flexible PVSC by SCM, China.

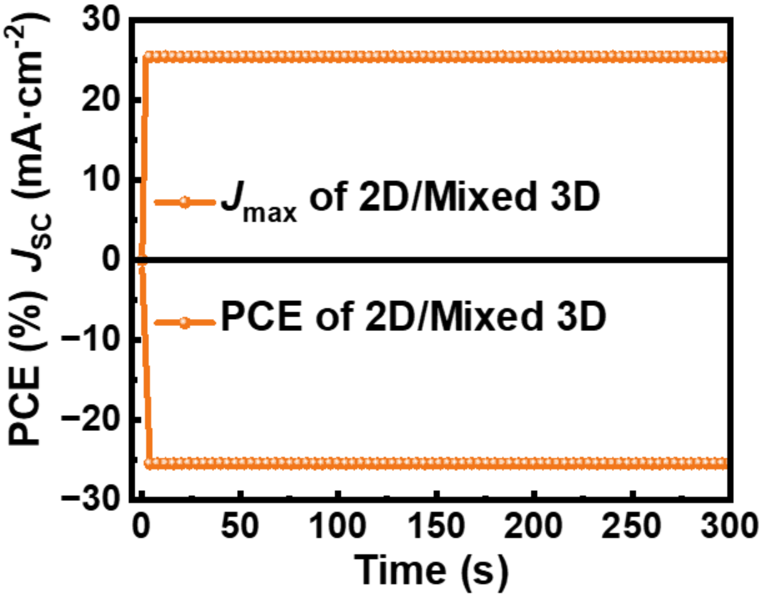

Fig. S34. SPO of flexible PVSC based on 2D/mixed 3D perovskite film.

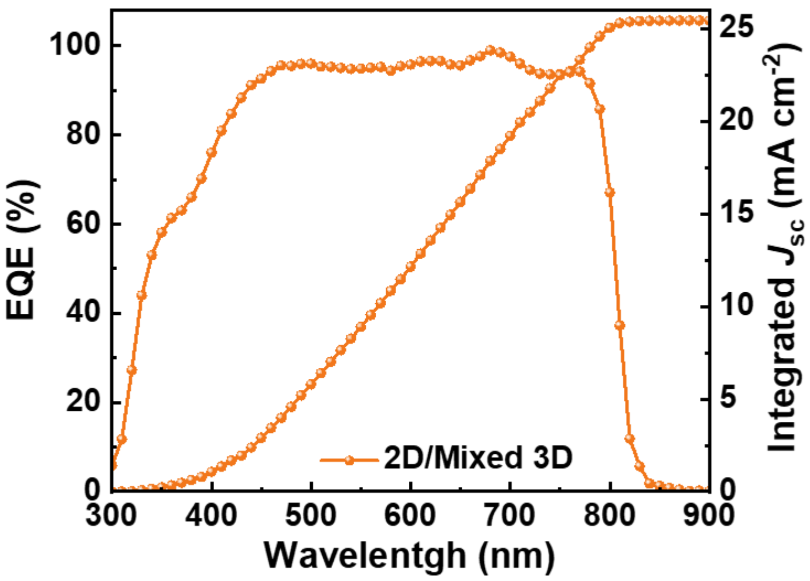

Fig. S35. EQE for flexible PVSCs based on 2D/mixed 3D perovskite films.

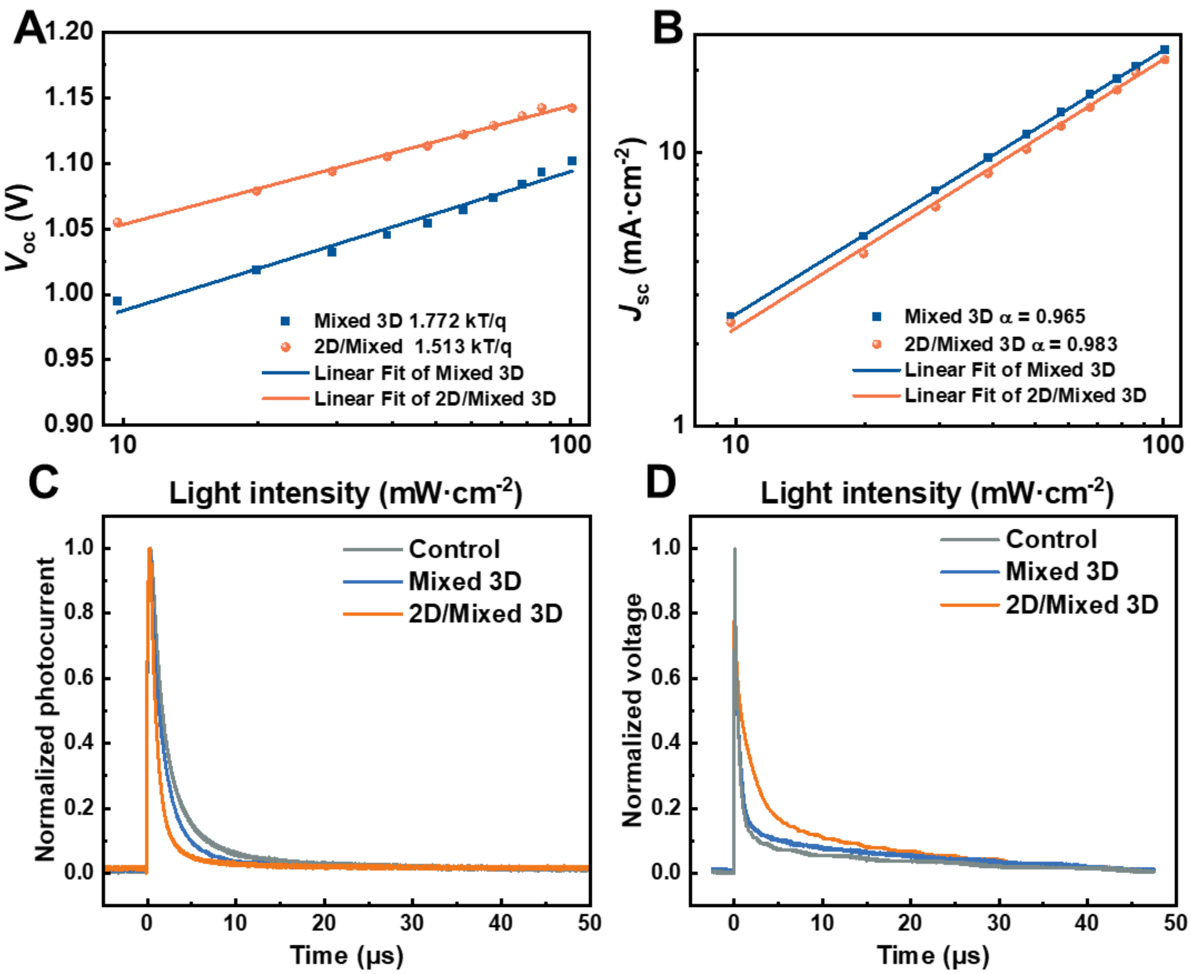

Fig. S36. Light intensity dependence of *V*oc and *J*sc, TPV and TPC for flexible PVSCs.

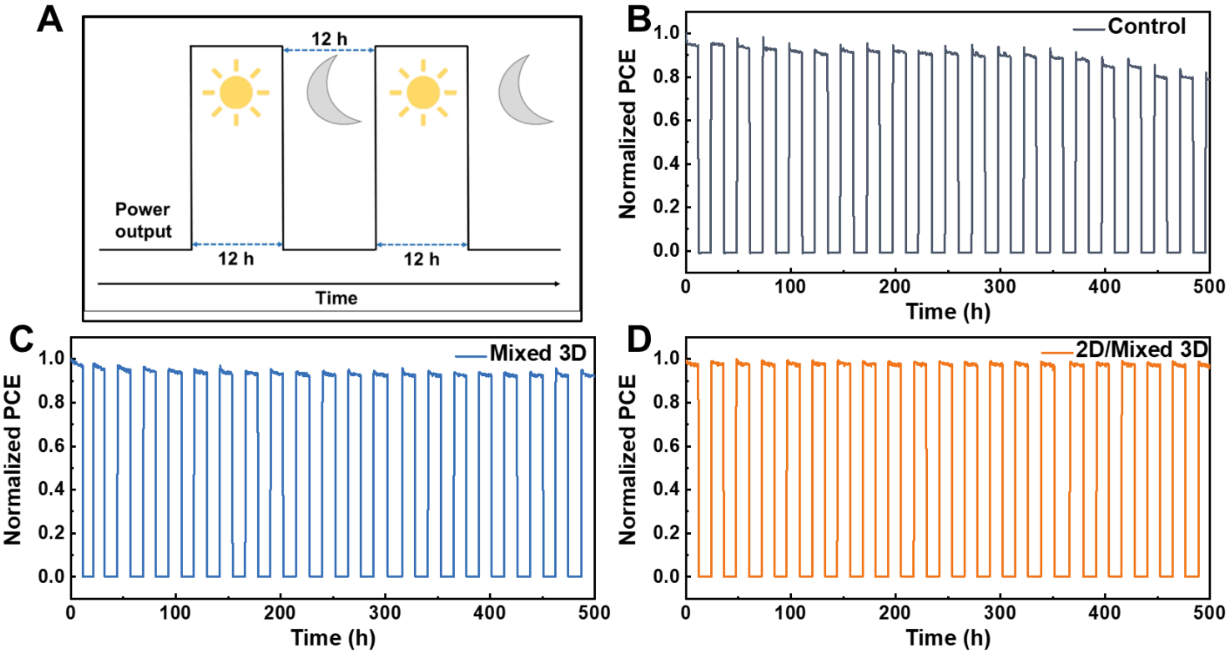

Fig. S37. Day/night cycling stability tests. Normalized evolution of PCE for unencapsulated flexible PVSCs based on control, mixed 3D, and 2D/mixed 3D perovskite films under periodic day/night cycling conditions (12 h under 1-sun illumination at ~55 °C, followed by 12 h in the dark at ~25 °C).

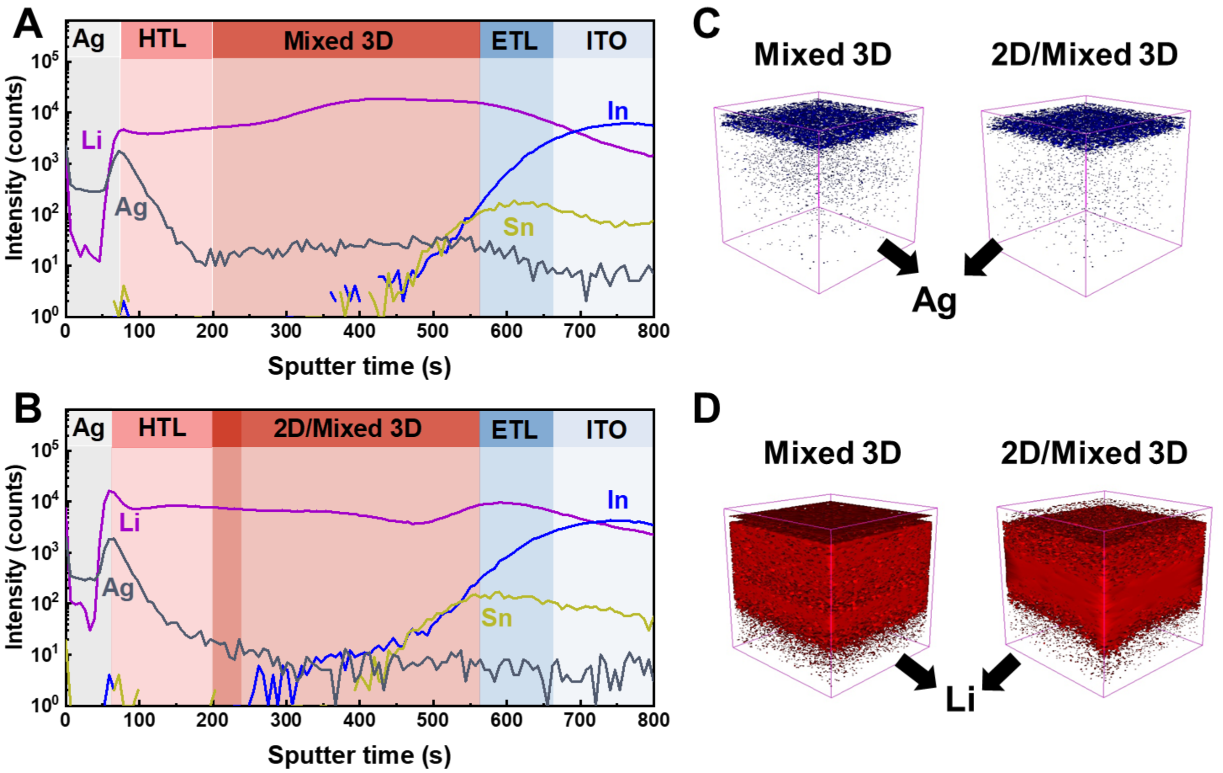

Fig. S38. TOF-SIMS analysis. TOF-SIMS spectra and corresponding 3D render overlay images with Ag^{+} and Li^{+} of devices based on mixed 3D and 2D/mixed 3D perovskite layers after 85 °C for 500 h.

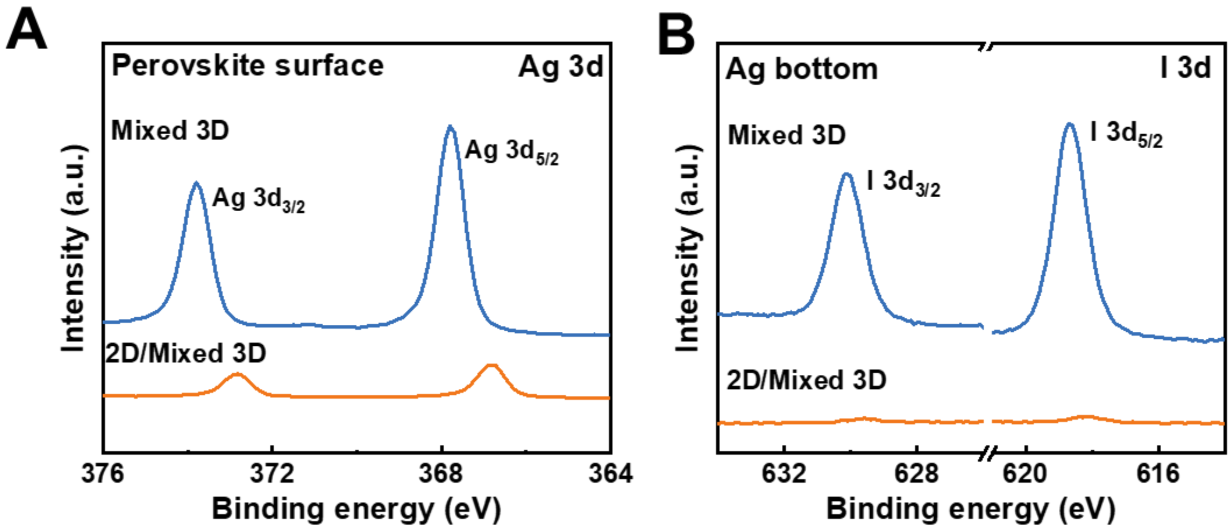

Fig. S39. XPS Ag 3d and I 3d spectra acquired on the exposed electrode bottom of devices based on mixed 3D and 2D/mixed 3D films following aging at 85 °C for 500 h.

Surface-sensitive XPS analysis of the perovskite film (after carefully removing the top contacts) revealed a markedly attenuated Ag 3d signal on the surface of the 2D/mixed 3D sample compared to the control. This directly confirms that less Ag has diffused to the perovskite surface, corroborating the TOF-SIMS findings at the interface. The 2D/mixed 3D device shows a negligible I 3d signal at this interface, in stark contrast to the pronounced iodine peak detected in the mixed 3D control device. This confirms that significantly less iodine has migrated through the 2D-capped film to reach and corrode the electrode.

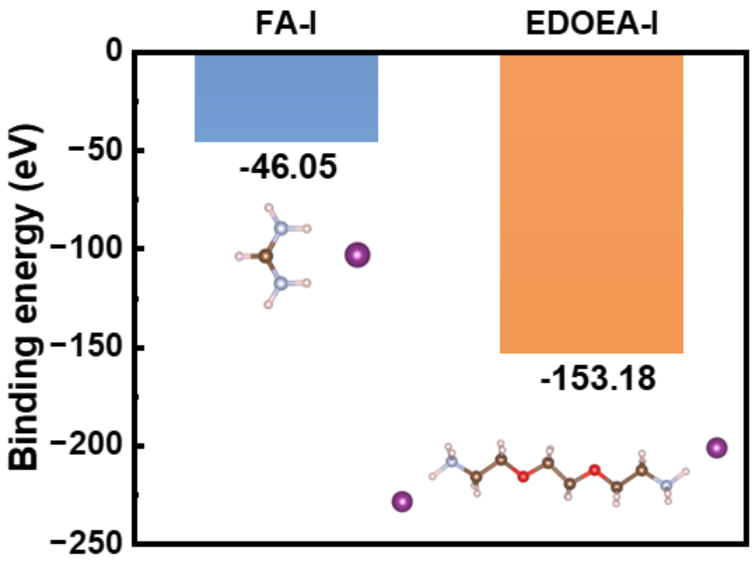

Fig. S40. Binding energy of FA-I and EDOEA-I.

The binding energy between the EDOEA spacer cation and an iodide ion (I^-) is -153.18 eV, significantly stronger than that between the formamidinium (FA^+) cation and I^- (-46.05 eV) in the 3D bulk. This indicates the 2D structure provides a thermodynamically favorable site to anchor iodide.

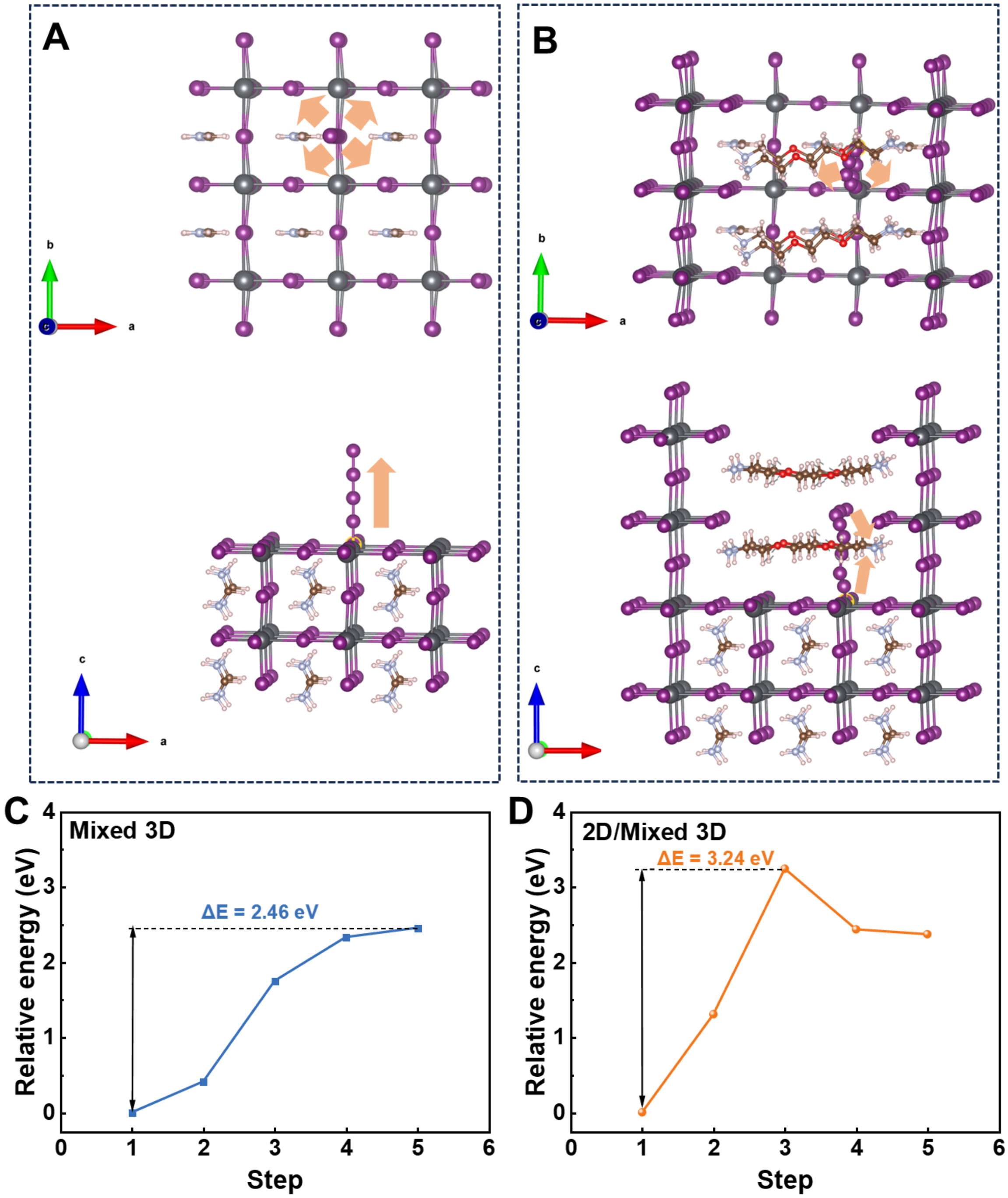

Fig. S41. Iodine migration energy barrier in mixed 3D and 2D/mixed 3D perovskites. (**A, B**) model structure (top view and side view) and (**C, D**) calculated energy barrier of the diffusion of iodine migration in mixed 3D perovskite and 2D/mixed 3D perovskite.

The energy barrier for iodine migration within the 2D/mixed 3D perovskite is calculated to be 3.24 eV, which is ~32% higher than in the mixed 3D perovskite (2.46 eV). This increased activation energy directly translates to a slower iodine diffusion rate.

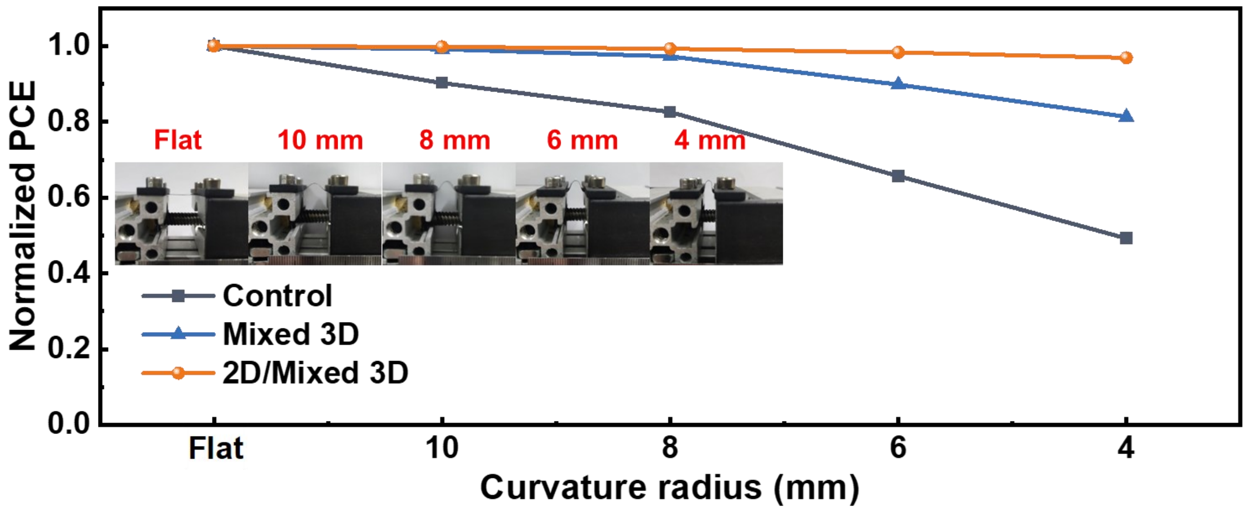

Fig. S42. Normalized PCE values of flexible PVSCs after bending 1000 cycles with different bending radii.

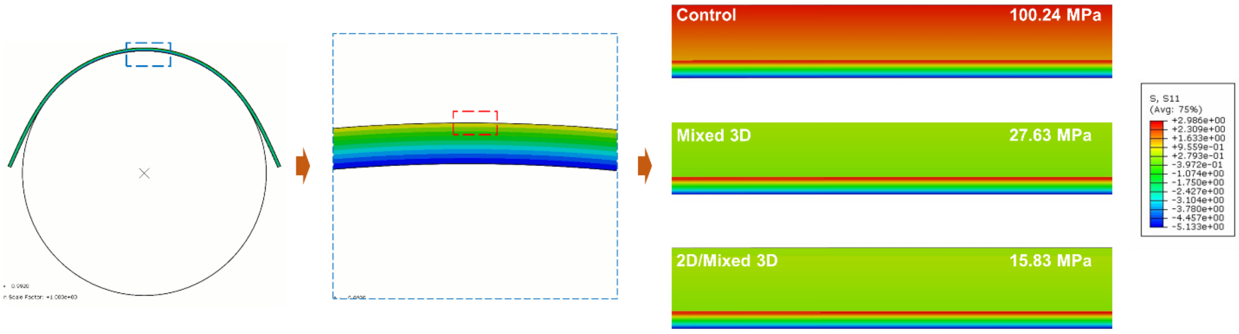

Fig. S43. Finite element analysis (FEA) of the stress distribution in the device under bending conditions. The bending simulations were conducted using a 2D plane-strain model in ABAQUS/Standard to replicate the experimental bending condition. The multilayer film stack was discretized using 8-node quadratic plane strain elements (CPE8R). A global element size of 150 µm was used, with local refinement at the perovskite and interfacial layers to ensure convergence of stress gradients. The cylindrical mandrel was modeled as a rigid body using 2-node rigid link elements (R2D2). The complete mesh comprised 59,994 elements and 193,333 nodes for the film stack, and 9,308 elements with 9,309 nodes for the mandrel. The boundary conditions fixed one end of the substrate (PEN/ITO), while the rigid mandrel was displaced to impose a 4 mm bending radius, with frictionless hard contact defined between the mandrel and the device surface.

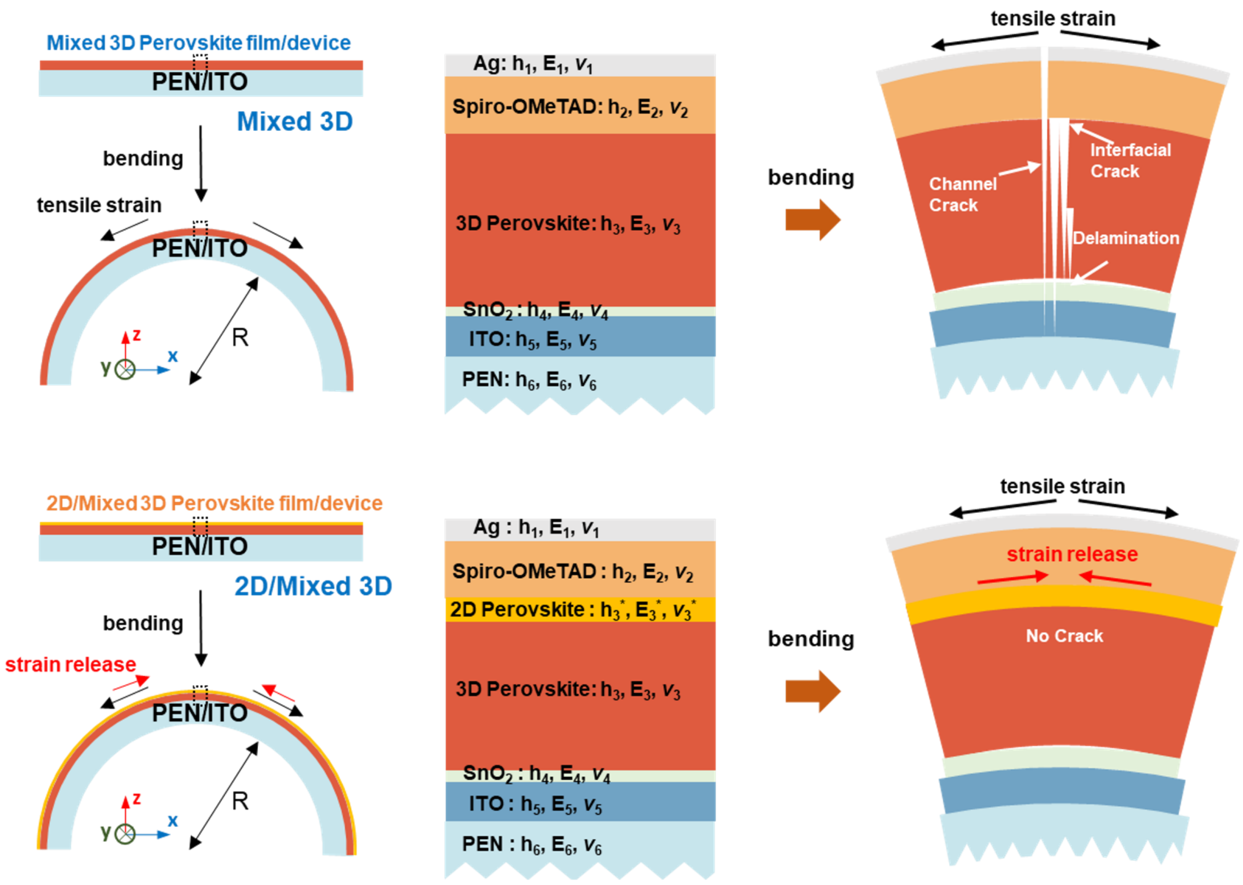

Fig. S44. Schematic illustration of the flexible PVSC bending test setup, detailing the 6-layer configuration used for finite element method simulations to model mechanical behavior. The FEM simulations are used to replicate the experimental bending conditions, and the stress distribution and failure mechanisms are highlighted, showing how the 2D/mixed 3D configuration reduces peak stress and delays crack initiation. The material properties used for the FEM simulations are summarized in **Table S10**.

Table S1. The summary of Young's modulus measured by PF-QNM-AFM and nanoindentation.

Sample	Young's modulus measured by PF-QNM-AFM (GPa)	Young's modulus measured by nanoindentation (GPa)
Control	8.54	38.24
Mixed 3D w/ Ace	6.50	30.52
Mixed 3D w/ GA	3.16	26.98
Mixed 3D w/ MDA	2.34	21.72

Table S2. Defect formation energies of $FAPbI_3$ perovskite systems with different A-site cation mixtures with the negatively charged defects, including iodine-lead anti-sites (I_{Pb}), formamidinium vacancies (V_{FA}), lead vacancies (V_{Pb}), iodine-formamidinium anti-sites (I_{FA}) and iodine interstitials (I_i).

defects	FA	Ace	GA	MDA
I_{Pb}	-3.86	-5.27	-5.87	-8.83
V_{FA}	-4.14	-5.26	-5.69	-9.54
V_{Pb}	-3.98	-5.38	-6.73	-9.82
I_{FA}	-2.49	-4.6	-5.2	-8.15
I_i	-3.37	-3.45	-4.42	-7.73

Table S3. Tolerance factors (*t*) calculated with different x values in ($FA_{0.93}MA_{0.07-x}A_xPbI_3$ ((here A means that A-site cations including Ace, GA and MDA).

x-value	0	1%	3%	5%	10%
t (Ace)	0.96013	0.960634	0.961623	0.962633	0.965138
t (GA)	0.96013	0.960655	0.961686	0.962739	0.965348
t (MDA)	0.96013	0.960318	0.960676	0.961055	0.961981

Goldschmidt tolerance factor: $t = \frac{r_A + r_X}{\sqrt{2}(r_B + r_X)}$

Table S4. Champion photovoltaic parameters of rigid PVSCs devices under standard AM 1.5 G illumination (100 mW·cm^{-2}).

Sample	V_{oc} (V)	J_{sc} (mA·cm^{-2})	FF (%)	PCE (%)
Control	1.171	25.41	78.39	23.34
Mixed 3D	1.176	26.10	82.12	25.20
2D/Mixed 3D	1.190	26.52	84.60	26.59

Table S5. Champion photovoltaic parameters of flexible PVSCs devices under standard AM 1.5 G illumination (100 mW·cm^{-2}).

Sample	Scan direction	V_{oc} (V)	J_{sc} (mA·cm^{-2})	FF (%)	PCE (%)	HI
Control	RS	1.169	24.51	77.74	22.28	0.0821
	FS	1.153	24.25	73.26	20.45	
Mixed 3D	RS	1.171	25.13	81.75	24.06	0.0254
	FS	1.161	24.94	81.01	23.45	
2D/Mixed 3D	RS	1.180	26.02	84.27	25.88	0.0089
	FS	1.174	25.96	84.18	25.65	

Hysteresis index factor: $HI = \frac{PCE_{RS} - PCE_{FS}}{PCE_{RS}}$

Table S6. Champion photovoltaic parameter of 5×5 cm^2 area flexible perovskite solar modules based on 2D/Mixed 3D w/ MDA perovskite film under standard AM 1.5 G illumination (100 $mW\cdot cm^{-2}$).

Flexible PVSM	V_{oc} (V)	J_{sc} ($mA\cdot cm^{-2}$)	FF (%)	PCE (%)
5 × 5 cm^2	8.12	3.70	72.32	21.77
10 × 10 cm^2	11.46	2.34	71.93	19.23

Table S7. Summary of recently reported flexible PVSCs and PVSMs with high efficiency.

Device structure	Device architecture	V_{oc} (V)	J_{sc} ($mA\cdot cm^{-2}$)	FF (%)	PCE (%)	Active area (cm^2)	Ref.
**n-i-p**	**PEN/ITO/SnO_2/$FA_{0.93}MA_{0.04}MDA_{0.03}PbI_3$/ $EDOEAI_2$/Spiro-OMeTAD/Ag**	**1.18**	**26.02**	**84.27**	**25.88**	**0.08**	**This work**
		**8.12**	**3.70**	**72.32**	**21.77**	**16.9**	
		**11.46**	**2.34**	**71.93**	**19.23**	**78.2**	
p-i-n	PEN/ITO/PTAA/$MA_{0.7}FA_{0.3}PbI_3$/C_{60}/BCP/Cu	1.18	24.18	81.9	23.4	0.08	*Nat. Photon.* (2024) (*68*)
		5.85	4.26	75.48	18.8	9	
n-i-p	PEN/ITO/$SrSnO_3$/$FACsPbI_3$/OATsO/ Spiro-OMeTAD/Au	1.15	23.65	81.2	22.08	0.049	*Nat. Photon.* (2023) (*73*)
n-i-p	PET/ITO/SnO_2/$FAPbI_3$/PEAI/Spiro-OMeTAD/Au	1.177	25.73	83.31	25.23	0.08	*Nat. Photon.* (2025) (*54*)
p-i-n	PET/AlO_x/PEDOT:PSS/$MBA_2(Cs_{0.12}MA_{0.88})_6Pb_7I_{22}$/ PCBM/$TiO_x$/Cr/Au	1.15	22.4	78.0	20.1	0.9	*Nat. Energy* (2024) (*12*)
p-i-n	PET/ITO/NiOx/PTAA/ $Cs_{0.05}(FA_{0.95}MA_{0.05})_{0.95}Pb(I_{0.95}Br_{0.05})_3$/PCBM/BCP/Ag	1.172	23.89	84.72	23.72	0.0242	*Nat. Commun.* (2025) (*60*)
n-i-p	PET/ITO/SnO_2/$FA_{0.92}MA_{0.08}PbI_{2.76}Br_{0.24}$/ Spiro-OMeTAD/Au	1.174	25.45	81.74	24.43	0.045	*Nat. Commun.* (2025) (*49*)
n-i-p	PEN/ITO/SnO_2/$Cs_{0.04}(FA_{0.84}MA_{0.16})_{0.96}Pb(I_{0.84}Br_{0.16})_3$/ Spiro-OMeTAD/Au	1.15	22.4	78.2	20.1	0.16	*Nat. Commun.* (2021) (*82*)
n-i-p	PEN/ITO/SnO_2/$Cs_{0.05}FA_{0.85}MA_{0.1}PbI_3$/ Spiro-OMeTAD/Au	1.15	24.69	78.0	22.1	0.16	*Nat. Commun.* (2023) (*11*)
n-i-p	PET/ITO/SnO_2/HBPs/$(FAPbI_3)_{1-x}(MAPbBr_3)_x$/ Spiro-OMeTAD/Au	1.177	25.07	80.83	23.86	0.045	*Nat. Commun.* (2023) (*66*)
p-i-n	PEN/ITO/4PADCB/ $Cs_{0.05}(FA_{0.96}MA_{0.04})_{0.95}(I_{0.96}Br_{0.04})_3$/$C_{60}$/BCP/Ag	1.166	25.35	83.4	24.64	0.04	*Sci. Adv.* (2025) (*9*)
		5.584	4.40	69.7	17.13	10.24	
n-i-p	PEN/ITO/SnO_2/$FAPbI_3$/c-HAI/Spiro-OMeTAD/Ag	1.20	24.91	81.79	24.51	0.0813	*Joule* (2024) (*57*)
n-i-p	PEN/ITO/SnO_2/$FA_{0.92}MA_{0.08}PbI_3$/Spiro-OMeTAD/Au	1.14	25.1	81.79	23.4	0.062	*Joule* (2023) (*69*)
n-i-p	PEN/ITO/SnO_2/$FAPbI_3$/Spiro-OMeTAD/Au	1.16	22.3	83.6	22.92	0.094	*Joule* (2024) (*71*)
		11.23	1.91	74.6	16.0	25	
		54.0	0.488	62.36	16.43	900	
n-i-p	PET/ITO/TMACL-SnO_2/$FACsPbI_3$/OAmBr/ Spiro-OMeTAD/Au	1.20	24.67	79.5	23.54	0.1	*Joule* (2025) (*67*)
		19.81	1.31	77.24	20.08	57.20	
n-i-p	PET/ITO/TiO_2/$(4AP)PbI_4$/$FAPbI_3$/ Spiro-OMeTAD/Au	1.15	25.26	76.58	22.3	0.09	*Joule* (2023) (*72*)
n-i-p	PEN/ITO/SnO_2/$FA_{0.92}MA_{0.08}PbI_3$/Spiro-OMeTAD/Au	1.136	26.27	78.49	23.6	0.1	*The Innovation* (2022) (*61*)

n-i-p	PEN/ITO/SnO_2/$FAPbI_3$/Spiro-OMeTAD/Ag	1.177	24.69	84.16	24.46	0.04	*Angew. Chem. Int. Ed.* (2025) (*59*)
p-i-n	PEN/ITO/MPACPA/Perovskite/PEACl/ PMMA/C_{60}/BCP/Ag	1.21	25.49	82.03	25.3	0.05	*Angew. Chem. Int. Ed.* (2025) (*63*)
n-i-p	PET/ITO/SnO_2/Perovskite/Spiro-OMeTAD/Au	1.161	25.29	78.93	23.18	0.045	*Angew. Chem. Int. Ed.* (2025) (*70*)
n-i-p	PEN/ITO/SnO_2/FAMACsPbIBr/Spiro-OMeTAD/Ag	1.184	25.69	83.59	25.42	0.101	*Adv. Mater.* (2025) (*62*)
		9.938	2.254	75.85	16.99	100	
n-i-p	PET/ITO/SnO_2/$(FAPbI_3)_{1-x}(MAPbBr_3)_x$/ Spiro-OMeTAD/Au	1.20	24.62	81.73	24.15	0.09	*Adv. Mater.* (2025) (*56*)
		8.38	1.99	79.21	21.82	12.80	
n-i-p	PEN/ITO/SnO_2/$FAPbI_3$/PEAI/Spiro-OMeTAD/Ag	1.17	25.47	81.08	24.19	0.07	*Adv. Mater.* (2025) (*58*)
n-i-p	PEN/ITO/SnO_2/$Cs_{0.05}MA_{0.05}FA_{0.9}PbI_3$/ PEAI/Spiro-OMeTAD/Au	1.161	25.88	82.7	24.85	0.060	*Adv. Mater.* (2024) (*65*)
		8.59	3.27	77.08	21.65	15.82	
n-i-p	PEN/ITO/SnO_2/FAMACsPbIBr/Spiro-OMeTAD/Ag	1.191	25.50	83.98	25.55	1.01	*Adv. Funct. Mater.* (2025) (*64*)
		12.26	2.142	62.43	16.39	100	

Table S8. Summary of long-term stability (measured according to ISOS-L-3 protocol), damp-heat conditions (85 °C and 85% relative humidity) following the ISOS-D-3 protocol, temperature cycling tests from -40 °C to 85 °C of recently reported flexible PVSCs.

Device structure	Device architecture	PCE (%)	Stability	Ref.
**n-i-p**	**PET/ITO/SnO_2/$FA_{0.93}MA_{0.04}MDA_{0.03}PbI_3$/ $EDOEAI_2$/Spiro-OMeTAD/Ag**	**25.88**	**ISOS-L-1, MPP, N_2, 2000 h-maintained 97.8%**<br>**ISOS-D-3, encapsulated devices, 85 °C, 85% RH, 1000 h-maintained 89.9%**<br>**Temperature cycling tests from -40 °C to 85 °C, encapsulated devices, 500 cycles-maintained 92.1%**	**This work**
p-i-n	PET/AlOx/PEDOT:PSS/ $MBA_2(Cs_{0.12}MA_{0.88})_6Pb_7I_{22}$/PCBM/$TiO_x$/Cr/Au	20.1	ISOS-L-1, MPP, N_2, 1000 h-maintained 97%	*Nat. Energy* (2024) (*12*)
p-i-n	PET/ITO/NiOx/PTAA/$Cs_{0.05}(FA_{0.95}MA_{0.05})_{0.95}$ $Pb(I_{0.95}Br_{0.05})_3$/PCBM/BCP/Ag	23.72	ISOS-L-1, MPP, N_2, 800 h-maintained 95%	*Nat. Commun.* (2025) (*60*)
n-i-p	PET/ITO/SnO_2/$FA_{0.92}MA_{0.08}PbI_{2.76}Br_{0.24}$/ Spiro-OMeTAD/Au	24.43	ISOS-L-1, MPP, N_2, 1000 h-maintained 90%	*Nat. Commun.* (2025) (*49*)
n-i-p	PEN/ITO/SnO_2/$Cs_{0.05}FA_{0.85}MA_{0.1}PbI_3$/ Spiro-OMeTAD/Au	22.1	ISOS-L-1, MPP, N_2, 1443 h-maintained 85%	*Nat. Commun.* (2023) (*11*)
p-i-n	PEN/ITO/4PADCB/ $Cs_{0.05}(FA_{0.96}MA_{0.04})_{0.95}(I_{0.96}Br_{0.04})_3$/$C_{60}$/BCP/Ag	24.64	ISOS-L-1, MPP, N_2, 1000 h-maintained 91.8%	*Sci. Adv.* (2025) (*9*)
n-i-p	PEN/ITO/SnO_2/$FAPbI_3$/c-HAI/ Spiro-OMeTAD/Ag	24.51	ISOS-L-1, MPP, N_2, 500 h-maintained 97.8%<br>ISOS-L-3, 65 °C, 85% RH, encapsulated devices, 300 h-maintained 85%<br>ISOS-D-3, encapsulated devices, 65 °C, 85% RH, 300 h-maintained 80%	*Joule* (2024) (*57*)
n-i-p	PEN/ITO/SnO_2/$FA_{0.92}MA_{0.08}PbI_3$/ Spiro-OMeTAD/Au	23.6	ISOS-L-1, MPP, N_2, 500 h-maintained 80%	*The Innovation* (2022) (*61*)
n-i-p	PEN/ITO/SnO_2/$FAPbI_3$/Spiro-OMeTAD/Ag	24.46	ISOS-L-1, MPP, N_2, 1500 h-maintained 84.8%	*Angew. Chem. Int. Ed.* (2025) (*59*)
n-i-p	PET/ITO/SnO_2/$(FAPbI_3)_{1-x}(MAPbBr_3)_x$/ Spiro-OMeTAD/Au	24.15	ISOS-L-1, MPP, N_2, 200 h-maintained 90%	*Adv. Mater.* (2025) (*56*)
n-i-p	PEN/ITO/SnO_2/$FAPbI_3$/PEAI/ Spiro-OMeTAD/Ag	24.19	ISOS-L-1, MPP, N_2, 1300 h-maintained 93%	*Adv. Mater.* (2025) (*58*)
n-i-p	PEN/ITO/$Cs_{0.05}MA_{0.05}FA_{0.9}PbI_3$/ PEAI/Spiro-OMeTAD/ Au	24.85	ISOS-L-1, MPP, N_2, 1000 h-maintained 90%	*Adv. Mater.* (2024) (*65*)

Table S9. Summary of bending stability of flexible PVSCs with different bending radius in recent years.

Device structure	Device architecture	PCE (%)	bending radius (mm)	bending stability	Ref.
**n-i-p**	**PET/ITO/SnO_2/$FA_{0.93}MA_{0.04}MDA_{0.03}PbI_3$/ $EDOEAI_2$/Spiro-OMeTAD/Ag**	**25.88**	**4**	**11000 cycles, maintained 90%**	**This work**
p-i-n	PEN/ITO/PTAA/$MA_{0.7}FA_{0.3}PbI_3$/ C_{60}/BCP/Cu	23.4	20	5000 cycles, maintained 84%	*Nat. Photon.* (2024) (*68*)
n-i-p	PET/ITO/SnO_2/$FAPbI_3$/ PEAI/Spiro-OMeTAD/Au	25.23	5	1000 cycles, maintained 95.9%	*Nat. Photon.* (2025) (54)
p-i-n	PET/AlOx/PEDOT:PSS/ $MBA_2(Cs_{0.12}MA_{0.88})_6Pb_7I_{22}$/PCBM/TiOx/Cr/Au	20.1	0.1	100 cycles, maintained 99%	*Nat. Energy* (2024) (*12*)
p-i-n	PET/ITO/NiOx/PTAA/$Cs_{0.05}(FA_{0.95}MA_{0.05})_{0.95}Pb$ $(I_{0.95}Br_{0.05})_3$/PCBM/BCP/Ag	23.72	5	10000 cycles, maintained 86%	*Nat. Commun.* (2025) (*60*)
n-i-p	PET/ITO/SnO_2/$FA_{0.92}MA_{0.08}PbI_{2.76}Br_{0.24}$/ Spiro-OMeTAD/Au	24.43	10	10000 cycles, maintained 94.1%	*Nat. Commun.* (2025) (*49*)
n-i-p	PEN/ITO/SnO_2/ $Cs_{0.04}(FA_{0.84}MA_{0.16})_{0.96}Pb(I_{0.84}Br_{0.16})_3$/Spiro-OMeTAD/Au	20.1	3	2500 cycles, maintained 85%	*Nat. Commun.* (2021) (*82*)
n-i-p	PEN/ITO/SnO_2/$Cs_{0.05}FA_{0.85}MA_{0.1}PbI_3$/ Spiro-OMeTAD/Au	22.1	4	5000 cycles, maintained 86%	*Nat. Commun.* (2023) (*11*)
n-i-p	PET/ITO/SnO_2/HBPs/ $(FAPbI_3)_{1-x}(MAPbBr_3)_x$/Spiro-OMeTAD/Au	23.86	3	10000 cycles, maintained 88.9%	*Nat. Commun.* (2023) (*66*)
p-i-n	PEN/ITO/4PADCB/ $Cs_{0.05}(FA_{0.96}MA_{0.04})_{0.95}(I_{0.96}Br_{0.04})_3$/$C_{60}$/BCP/Ag	24.64	5	10000 cycles-maintained 91%	*Sci. Adv.* (2025) (*9*)
n-i-p	PEN/ITO/SnO_2/$FAPbI_3$/c-HAI/ Spiro-OMeTAD/Ag	24.51	2	10000 cycles, maintained 91%	*Joule* (2024) (*57*)
n-i-p	PEN/ITO/SnO_2/$FA_{0.92}MA_{0.08}PbI_3$/ Spiro-OMeTAD/Au	23.4	5	10000 cycles, maintained 86%	*Joule* (2023) (*69*)
n-i-p	PET/ITO/TiO_2/$(4AP)PbI_4$/$FAPbI_3$/ Spiro-OMeTAD/Au	22.3	2	3000 cycles, maintained 92%	*Joule* (2023) (*72*
n-i-p	PEN/ITO/SnO_2/$FA_{0.92}MA_{0.08}PbI_3$/ Spiro-OMeTAD/Au	23.6	6	10000 cycles, maintained 85%	*The Innovation* (2022) (*61*)
n-i-p	PEN/ITO/SnO_2/$FAPbI_3$/Spiro-OMeTAD/Ag	24.46	3	8000 cycles, maintained 84.65%	*Angew. Chem. Int. Ed.* (2025) (*59*)
p-i-n	PEN/ITO/MPACPA/Perovskite/PEACl/PMMA/ C_{60}/BCP/Ag	25.3	10	5000 cycles, maintained 95.3%	*Angew. Chem. Int. Ed.* (2025) (*63*)
n-i-p	PET/ITO/SnO_2/Perovskite/Spiro-OMeTAD/Au	23.18	3	10000 cycles, maintained 96%	*Angew. Chem. Int. Ed.* (2025) (*70*)
n-i-p	PEN/ITO/$Cs_{0.05}MA_{0.05}FA_{0.9}PbI_3$/PEAI/ Spiro-OMeTAD/Au	24.85	3	5000 cycles, maintained 94.8%	*Adv. Mater.* (2024) (*65*)

Table S10. The thicknesses and elastic properties of the seven layers in the flexible PVSCs used for the FEM modeling.

Layer # i	Material	Thickness h_i (nm)	Young's Modulus E_i (GPa)	Poisson's Ratio v_i
1	Ag	100	19.5	0.30
2	Spiro-OMeTAD	200	0.5	0.45
3	DJ 2D perovskite	20	1.34	0.40
4	3D perovskite	700	8.54 (Control)<br>2.34 (Mixed 3D)	0.33
5	SnO_2	20	150	0.25
6	ITO	200	190	0.25
7	PEN	125	5	0.45
